# Identification and validation of tumor-infiltrating lymphocyte-related prognosis signature for predicting prognosis and immunotherapeutic response in bladder cancer

**DOI:** 10.1186/s12859-023-05241-z

**Published:** 2023-03-27

**Authors:** Canxuan Li, Weibin Xie

**Affiliations:** 1 Department of Urology, Shenshan Medical Center, Memorial Hospital of Sun Yat-Sen University, Shanwei, Guangdong People’s Republic of China; 2 Department of Urology, Sun Yat-Sen Memorial Hospital, Sun Yat-Sen University, Guangzhou, Guangdong People’s Republic of China; 3 Guangdong Provincial Key Laboratory of Malignant Tumor Epigenetics and Gene Regulation, Sun Yat-Sen Memorial Hospital, Sun Yat-Sen University, Guangzhou, Guangdong People’s Republic of China; 4 Guangdong Provincial Clinical Research Center for Urological Diseases, Guangzhou, Guangdong People’s Republic of China

**Keywords:** Tumor-infiltrating lymphocytes, Bladder cancer, Immunotherapy response, Prognostic signature

## Abstract

**Background:**

It has been discovered that tumor-infiltrating lymphocytes (TILs) are essential for the emergence of bladder cancer (BCa). This study aimed to research TIL-related genes (TILRGs) and create a gene model to predict BCa patients' overall survival.

**Methods:**

The RNA sequencing and clinical data were downloaded from the TGCA and GEO databases. Using Pearson correlation analysis, TILRGs were evaluated. Moreover, hub TILRGs were chosen using a comprehensive analysis. By dividing the TCGA-BCa patients into different clusters based on hub TILRGs, we were able to explore the immune landscape between different clusters.

**Results:**

Here, we constructed a model with five hub TILRGs and split all of the patients into two groups, each of which had a different prognosis and clinical characteristics, TME, immune cell infiltration, drug sensitivity, and immunotherapy responses. Better clinical results and greater immunotherapy sensitivity were seen in the low-risk group. Based on five hub TILRGs, unsupervised clustering analysis identify two molecular subtypes in BCa. The prognosis, clinical outcomes, and immune landscape differed in different subtypes.

**Conclusions:**

The study identifies a new prediction signature based on genes connected to tumor-infiltrating lymphocytes, providing BCa patients with a new theoretical target.

**Supplementary Information:**

The online version contains supplementary material available at 10.1186/s12859-023-05241-z.

## Introduction

Bladder cancer is the second most common type of urologic cancer, presenting as a non-muscle invasive lesion (NMIBC) in 70% of cases [1, 2]. About 25–75% of high-risk NMIBCs develop into muscle-invasive cancer (MIBC) and later metastatic cancer, with a terrible prognosis [3, 4]. Even with neoadjuvant and adjuvant chemotherapy, metastatic BCa has a poor prognosis [5]. For advanced BCa, cancer immunotherapy, such as immune checkpoint blockade (ICB), has shown promising survival advantages [6]. Due to the main or secondary mechanisms of resistance to ICB, only a small percentage of patients respond to it [7]. To enhance the prognosis of BCa patients, it is essential to develop innovative, focused therapy.

Human malignancies have a complicated composition [8]. Macrophages and conventional T cells dominate immune infiltration in the majority of malignancies [9]. According to conventional wisdom, tumor-infiltrating lymphocytes (TILs) are a diverse population of T cells, including CD4+ and CD8+ subpopulations, that are present in the tumor microenvironment (TME) [10]. It is universally accepted that all lymphoid immune system cells must be present, activated, and co-stimulated for an effective antitumor immune response ]^11^, ^12^]. TIL therapy is an adoptive cell therapy in which tumor-derived infiltrating lymphocytes are cultivated and increased in vitro before being reinfused into patients as a therapeutic agent [13]. In comparison to other adoptive cell therapies, TIL therapy has a distinct advantage in the treatment of solid tumors due to its diversified TCR clonality, superior tumor-homing ability, and reduced off-target toxicity [14]. TILs in primary bladder tumors and lymph nodes can be extracted from patients, multiplied, and show anti-tumor activity in vitro, according to a recent study [15]. Exploration of genes associated with TILs is therefore essential to comprehending BCa treatment and prognosis.

Herein, a TIL-related gene signature (TILRGS) was constructed by bioinformatics analysis for predicting cancer prognosis and treatment response in BCa. Specifically, TILs scores were first calculated for each patient using ssGSEA analysis, then TILRGs were obtained through Pearson correlation analysis, differentially expressed TILRGs between tumor and normal samples were identified with the "Limma" package, and key TILRGs were identified and prognostic models were built with univariate, LASSO and multifactorial Cox regression analysis. Moreover, we explored the association between BCa gene signatures and the immune microenvironment. The findings of this study will contribute to our understanding of the immune infiltration process of TILs in BCa, help us identify new immunotherapy targets, and improve the prognosis of BCa patients.

## Materials and methods

### Acquisition of public data

We gathered the clinical information and RNA sequencing data of 410 bladder tumor samples and 19 control bladder samples from The Cancer Genome Atlas database (TCGA, https://portal.gdc.cancer.gov/), from which we extracted basic information, clinical traits, and survival data (survival time and survival status). 404 bladder cancer tissue samples were eventually included in this study after duplicate samples and ones lacking survival time were eliminated. The Gene Expression Omnibus database (GEO, https://www.ncbi.nlm.nih.gov/geo/) yielded the GSE13507 dataset, which included 144 bladder cancer samples with survival data [16, 17]. On the other side, Copy Number Variation (CNV) data were provided by the UCSC Xena database (http://xena.ucsc.edu/).

### Detection of tumor-infiltrating-lymphocyte-related genes (TILRGs)

Using single-sample Gene Set Enrichment Analysis (ssGSEA) [18], the "GSVA" R package (Version 3.13) was used to measure the amount of TILs infiltration in each BCa sample. The association between the TILs scores and the levels of gene expression was examined using the Pearson correlation coefficient. Genes were defined as TILRGs with p < 0.001 and correlation coefficient > 0.5.

### Differential expression analysis and functional enrichment analysis

In the TCGA-BCa dataset, the "Limma" R package (Version 3.13) was used to select differentially expressed TILRGs between normal and tumor samples. False discovery rate (FDR) < 0.05 and |log2 fold change (FC) |> 1 were the filtering criteria. Then, with an adjusted P-value threshold of 0.05, we ran GO and KEGG analyses using the "clusterProfiler" R package (Version 3.13).

### Construction and verification of a risk model

The training and test subgroups in this study were randomly divided among 404 participants from the TCGA-Bca dataset at a ratio of 7:3. For further verification, we also used the TCGA entire dataset and the GSE13507 dataset as validation datasets. TILRGs connected to overall survival in the training set were found using univariate Cox regression analysis, with the P-value set at less than 0.05. The CNV frequencies for prognosis-relevant TILRGs were shown in a lollipop chart. The prognosis-relevant TILRGs’ locations on the chromosomes, were visualized using the R package "RCircos" (Version 1.2.2). The waterfall plots were produced using the "maftools" R package (Version 3.13). The screened genes were then further compressed using the least absolute shrinkage and selection operator (LASSO) regression and hub TILRGs were chosen using multivariate Cox analysis. Combining the analysis, we developed a prognostic risk score formula, which was determined as follows: Risk score = (gene 1 expression * correspondence coefficient) + … + (gene n expression * correspondence coefficient). The patients were split into high-TILRGS and low-TILRGS subgroups based on the risk score's median value. To examine the differences in overall survival (OS) between the two groups, Kaplan–Meier (K–M) curves were utilized. The prognostic prediction accuracy of the TILRGS was evaluated using the time-dependent receiver operating characteristic (ROC) curves [19]. Also, we validated the model's prediction ability on the TCGA testing cohort, the entire TCGA cohort, and the GSE13507 cohort.

### Clinical application of the TILRGS

We used univariate and multivariate Cox regression to assess the independence of the TILRGS in the TCGA data set. Additionally, we assessed the accuracy of the TILRGS in comparison to other clinical parameters using the time-dependent ROC curves. Next, we developed a nomogram for predicting overall survival based on independent factors using the "rms" R package (Version 6.5-0). Calibration plots and ROC curves were assessed for the nomogram's accuracy and consistency. To examine differences in clinical variables between various risk groups, Wilcoxon and Chi-square tests were performed. K–M curves were used to compare the overall survival of patients in the high-TILRGS and low-TILRGS groups in distinct clinical groups.

### Analysis of molecular mechanisms and signaling pathways

With |log2 FC |> 1 and FDR < 0.05, we screened differentially expressed genes in various risk groups. Then, GO, KEGG, and GSEA analyses were carried out using the “clusterProfiler” R package (Version 3.13). The GSVA analysis was performed using the "GSVA" package in R (Version 3.13).

### Analysis of the immune landscape

Herein, the ESTIMATE algorithm was used to assess the immune scores in TCGA-BCa [20]. The CIBERSORT algorithm was used to estimate the relative proportions of 22 immune cells in TCGA-BCa [21]. ssGSEA was used for quantifying immune activity in TCGA-BCa [18]. The Wilcoxon test was used to evaluate immune scores, immune-infiltrating cell proportions, and immune activity scores between the two risk groups.

### Chemotherapeutic agents and immunotherapy

We evaluated the drug sensitivity of typical chemotherapy-targeted drugs using the R "pRRophetic" package (Version 1.0) [22]. To evaluate the connection between drug response and hub TILRGs expression levels, Spearman correlation analysis was used through the CellMiner database [23] (https://discover.nci.nih.gov/cellminer/home.do), which gives us access to a variety of drugs that have been clinically studied and approved by the Food and Drug Administration (FDA). The immunophenoscore (IPS) of BCa samples was obtained from the Cancer Immunome Atlas (TCIA, https://tcia.at/) database [24]. We used the Wilcoxon test to compare drug sensitivity and IPS between the high-TILRGS and low-TILRGS groups.

### Single cell analysis

The Tumor Immune Single-Cell Hub (TISCH, http://tisch.comp-genomics.org/home/) is a database dedicated to the exploration of the tumor microenvironment (TME) through single-cell RNA sequencing (scRNA-seq). The BLCA_GSE130001 dataset was selected to examine the expression of key genes among different cell types and within the dataset, four major cell types were identified: endothelial, epithelial, fibroblasts, and myofibroblasts.

### Cluster analysis of hub TILRGs

Using the "ConsensusClusterPlus" R package (Version 3.13), all TCGA-BCa patients were split into the ideal number of clusters based on the expression of hub TILRGs. The differences in OS between different clusters were examined using K–M curves. The Chi-square test was used to contrast clinical traits between different clusters. The Wilcoxon test was additionally employed to evaluate risk scores, immune scores, immune-infiltrating cell proportions, and immune activity scores among the different clusters.

### Statistical analysis

Herein, R software (Version 4.1.2) was employed to perform statistical analyses. The P-value for statistically significant differences was set to 0.05 unless otherwise noted in the text.

## Results

### Differentially expressed TILRGs (DETILRGs)

**Fig. 1:**
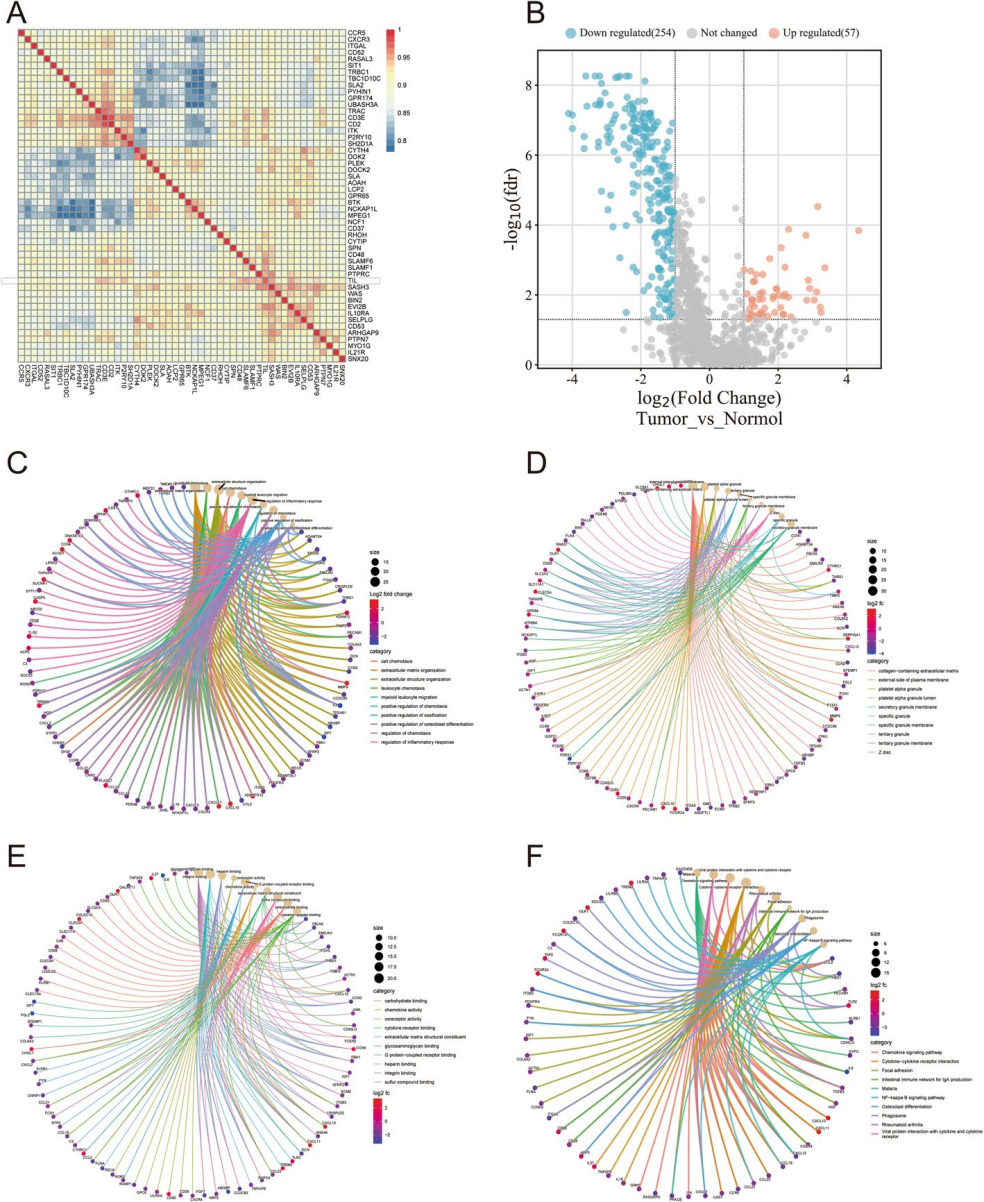
Identification of tumor-infiltrating lymphocyte-related genes (TILRGs). **a** The top 50 TILRGs’ correlations were performed using Spearmans correlation analysis; **b** Volcano plots showing differentially expressed TILRGs; **c**–**f** Gene ontology, including biological processes (BP), cellular components (CC), molecular functions (MF), and Kyoto Encyclopedia of Genes and Genomes (KEGG) analyses of differentially expressed TILRGs

In this investigation, a total of 1555 TILRGs were discovered (Additional file 1: Table S1). Figure 1a displayed the top 50 substantially associated genes. Following differential expression analysis, 311 DETILRGs were consequently screened (Fig. 1b). The findings of the GO and KEGG analyses were illustrated in Fig. 1c–f.

### Establishment and validation of a gene model

**Fig. 2:**
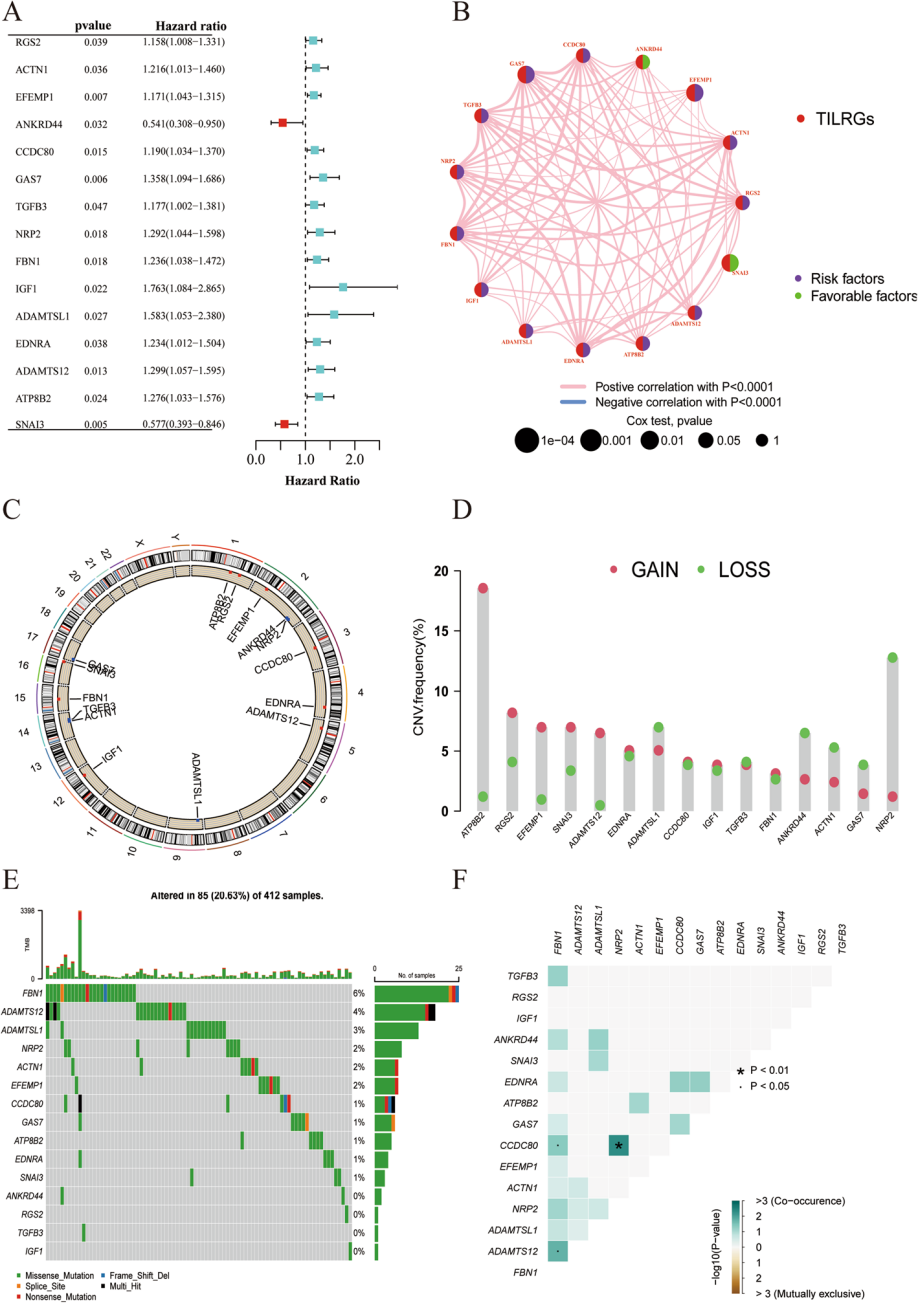
15 TILRGs were significantly associated with prognosis in the training cohort through univariate Cox regression analysis. **a** Forest plot of univariate Cox regression result; **b** Interaction between TILRGs in bladder cancer (BCa). The line connecting TILRGs indicated their interaction, and the thickness of the line indicated the correlation strength between TILRGs. Purple and green represent negative and positive correlation respectively; **c** The position of TILRGs copy number variant changes on 23 chromosomes; **d** Copy number variant mutation frequency of TILRGs; **e** The overall mutation profile of TILRGs in BCa; **f** Interaction effect of genes mutating differentially in BCa

**Fig. 3:**
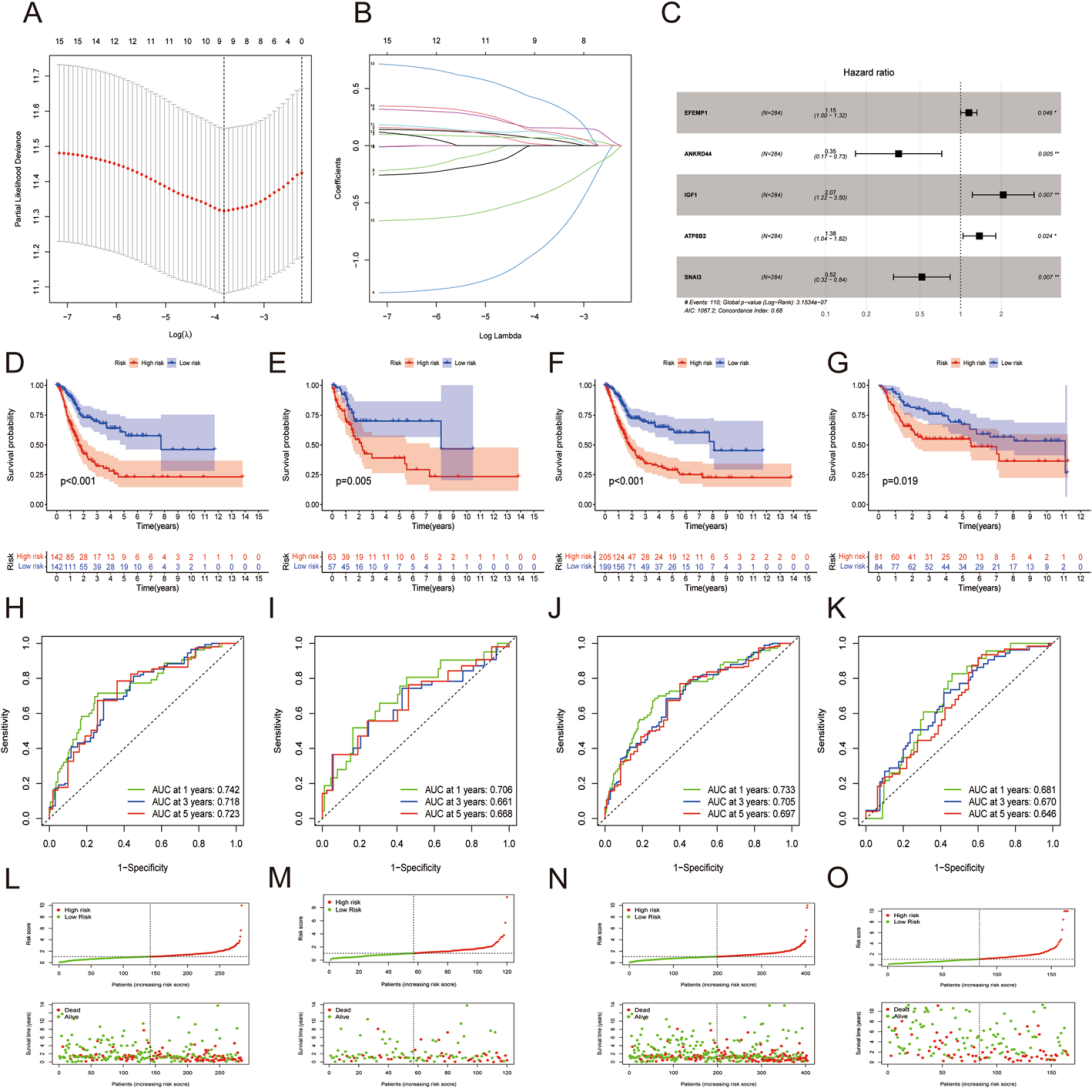
Development and validation of the tumor-infiltrating lymphocyte-related gene signature (TILRGS). **a**, **b** LASSO regression analysis; **c** Multivariate Cox model of five TILRGs; **d**–**g** The Kaplan–Meier curves in the TCGA-training cohort, TCGA-testing cohort, TCGA-all cohort and GEO-testing cohort, respective; **h**–**k** ROC analysis for OS prediction including 1, 3, and 5 years of BCa patients in the TCGA-training cohort, TCGA-testing cohort, TCGA-all cohort and GEO-testing cohort, respective; **l**–**o** Distribution of risk score, scatter plot of the overall survival of each patient, heat map of huh TILRGs expression level in the TCGA-training cohort, TCGA-testing cohort, TCGA-all cohort and GEO-testing cohort, respective. *P < 0.05; **P < 0.01

A total of 15 prognosis-related TILRGs were found in the training group by univariate Cox regression analysis (Fig. 2a), including 2 protective factors (ANKRD44, SNAI3) and 13 risk factors (RGS2, ACTN1, EFEMP1, CCDC80, GAS7, TGFB3, NRP2, FBN1, IGF1, ADAMTSL1, EDNRA, ADAMTS12, and ATP8B2). While the network plot shows the relationship between the expression levels of these TILRGs of rank much clearer (Fig. 2b). We further investigate the locations of these TILRGs on chromosomes as well as how they are altered (Fig. 2c, d). According to the findings, ATP8B2's most notable altered "gain" was found on chromosome 1, whereas NRP2's primary altered "loss" was found on chromosome 2. FBN1 was more commonly mutated when compared to other TILRGs in the TCGA BCa dataset, according to an oncoplot waterfall plot of the mutations and changes in these 15 TILRGs (Fig. 2e). Additionally, Fig. 2f demonstrated that only a few genes were altered simultaneously. Subsequently, the LASSO method was utilized to reduce the list of potential TILRGs and LASSO regression curves (Fig. 3a) and cross-validation plots (Fig. 3b) were obtained. Next, five hub TILRGs were identified using the Lasso and multivariate Cox analyses (Fig. 3c). The risk score was calculated as follows: Risk score = (0.141 × Exp EFEMP1) + (− 1.057 × Exp ANKRD44) + (0.727 × Exp IGF1) + (0.321 × Exp ATP8B2) + (− 0.660 × Exp SNAI3). The patients were separated into high-TILRGs and low-TILRGs groups. To better examine the prognostic significance of the risk signature using the median value of the risk score as the threshold. We discovered that in the TCGA-training, TCGA-testing, TCGA-all, and GEO-testing cohort, OS was considerably shorter in the high-TILRGs subgroup than in the low-TILRGs subgroup (Fig. 3d–g). In the TCGA-training cohort, the AUCs for the 1-, 3-, and 5-year OS were 0.742, 0.718, and 0.723, respectively (Fig. 3h). They were 0.706, 0.661, and 0.668 in the TCGA-testing cohort (Fig. 3i), and 0.733, 0.705, and 0.697 in the entire TCGA cohort (Fig. 3j), and 0.681, 0.670, and 0.646 in the GEO-testing cohort (Fig. 3k). In addition, we found that the proportion of dead patients was higher in the high-TILRGs subgroup than in the low- TILRGs subgroup (Fig. 3l–o).

### Evaluation of the clinical value of the TILRGS

**Fig. 4:**
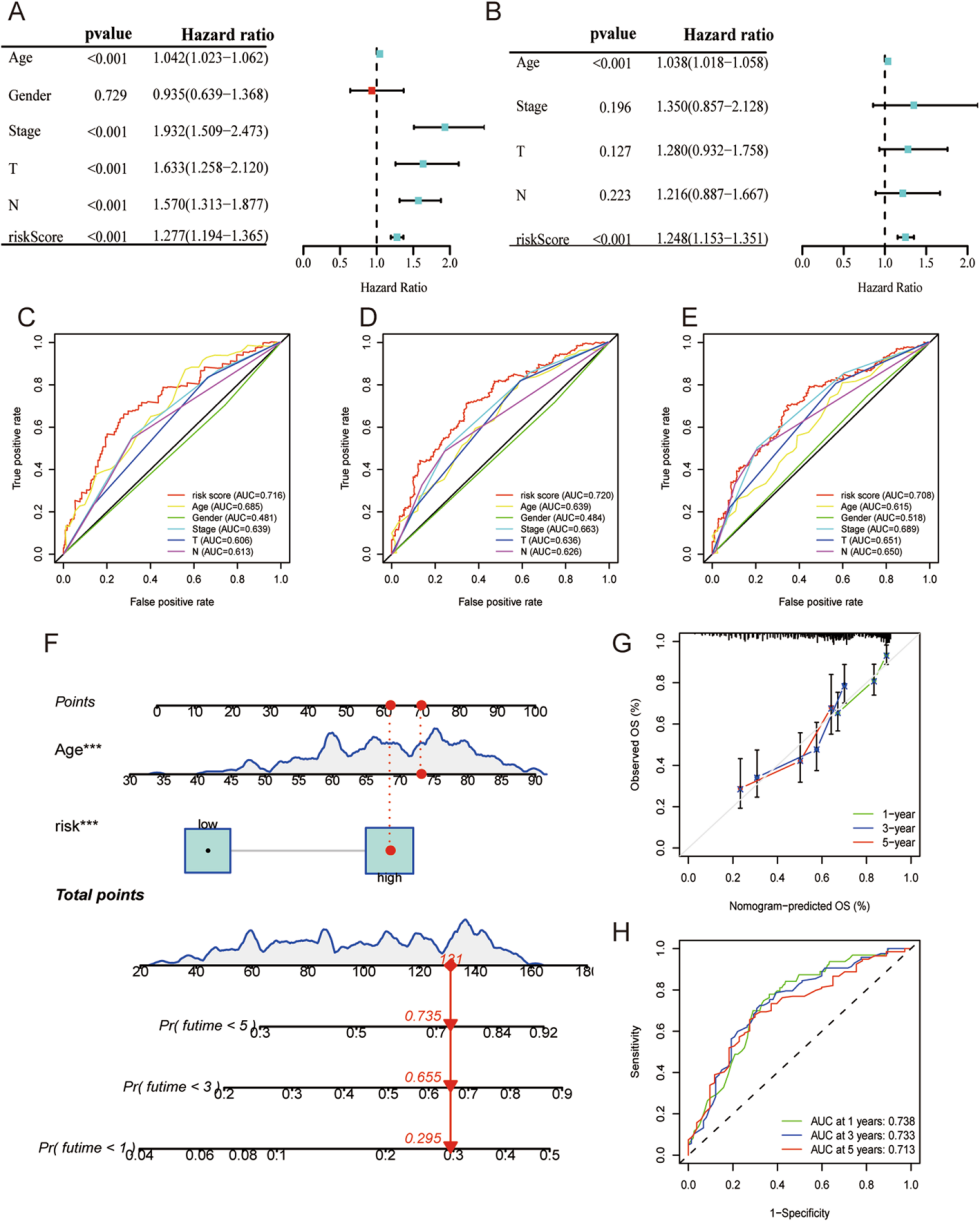
Establishment and assessment of the nomogram for survival prediction. **a**, **b** Univariate and multivariate Cox regression analyses showed that risk score is an independent prognostic factor affecting the prognosis of BCa patients in the TCGA dataset; **c**–**e** The 1-year, 3-year, and 5-year ROC curves of risk score and other clinicopathological parameters; **f** The nomogram of 1-year, 3-year, or 5-year OS; **g** Calibration curve for assess the agreement at 1-, 3- and 5-year OS; **h** The 1-year, 3-year, and 5-year ROC curves of nomogram

**Fig. 5:**
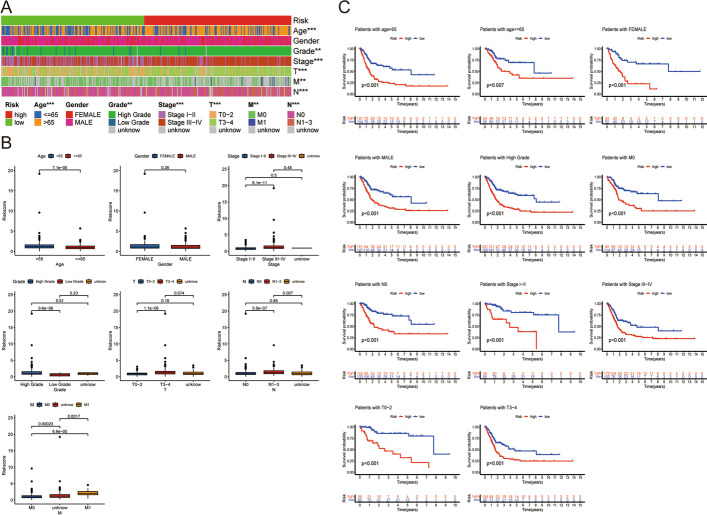
The gene signature was associated with the clinicopathological characteristics of patients with BCa in the TCGA dataset. **a** Heatmap for the gene signature and clinicopathological manifestation; **b** Boxplot of risk score in BCa patients with different clinical features; **c** Kaplan–Meier curves for the high- and low-risk groups stratified by clinical factors. N0, no lymph node metastasis; M0, No distant metastasis. **P < 0.01; ***P < 0.001

The risk score was an independent predictor of OS, according to our findings (Fig. 4a, b). Also, the AUCs value for 1-, 3-, and 5-year OS of the risk score was considerably greater than those of other clinical variables in the entire TCGA cohort (Fig. 4c–e). Then, we created a nomogram to forecast the overall survival with BCa (Fig. 4f). The forecast of the nomogram and the actual observation probabilities showed excellent agreement and accuracy through the calibration plots and ROC curves (Fig. 4g, h). We found significant correlations between risk score and clinical features except for gender in the entire TCGA cohort (Fig. 5a). Clinical traits, including age, pathologic grade, clinical tumor stage, and TNM status, were highly associated with the risk score (Fig. 5b). However, the risk score between male and female did not differ statistically significantly. Then, we divided the BCa patients into separate groups according to age (> 65 years and <  = 65 years), gender (male and female), tumor grade (high grade and low grade), pathological M stage (M0 and M1), pathological N stage (N0 and N1-3), T stage (T0-2 and T3-4), and pathological stage (I–II and III–IV) for further survival analysis. There were notable differences between the two risk groups in the majority of stratification categories, suggesting that the low-risk group had longer OS (Fig. 5c).

### Functional analyses based on the TILRGS

**Fig. 6:**
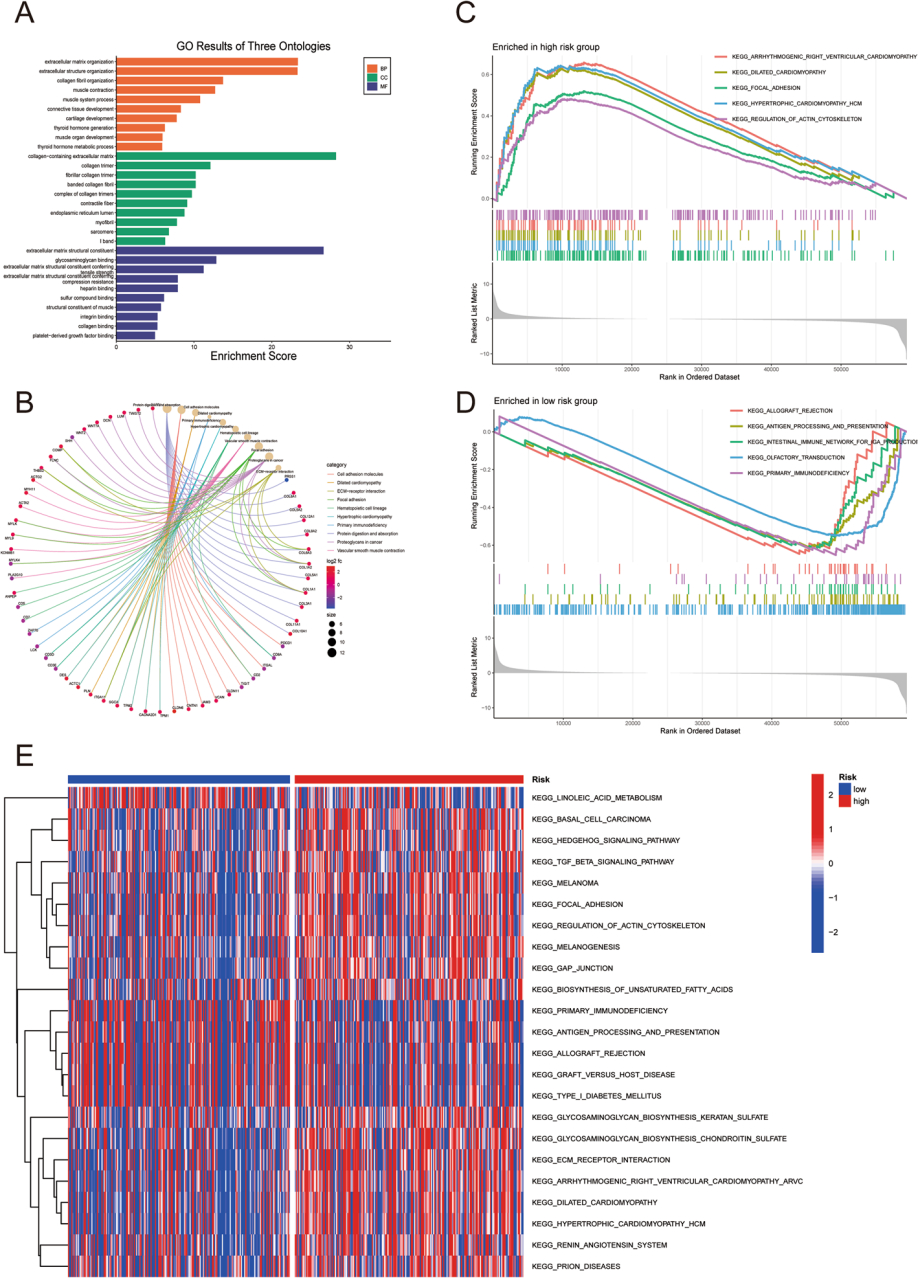
Functional analysis based on differentially expressed genes (DEGs) between the high-risk and low-risk groups. **a**, **b** Gene ontology and Kyoto Encyclopedia of Genes and Genomes (KEGG) analyses of DEGs; **c**, **d** Enrichment plots from gene set enrichment analysis in the high-risk group and low-risk group; **e** The heatmap displaying the signaling pathways in two risk subgroups analyzed by gene set variation analysis

Here, 305 DEGs between the various TILRGS subgroups were discovered (Additional file 1: Table S2). The findings of the GO and KEGG analyses performed on the aforementioned DEGs are displayed in Fig. 6a, b. The GSEA found that the high-TILRGS subgroup was enriched in arrhythmogenic right ventricular cardiomyopathy, dilated cardiomyopathy, focal adhesion, hypertrophic cardiomyopathy, and regulation of actin cytoskeleton (Fig. 6c), while the low-TILRGS subgroup was enriched in allograft rejection, antigen handling and presentation, the intestinal immune network for IgA production, olfactory transduction, and primary immunodeficiency (Fig. 6d). The GSVA enrichment analysis revealed that the enriched pathways between two TILRGS subgroup were different (Fig. 6e).

### Immune landscape of various TILRGS groups in BCa

**Fig. 7:**
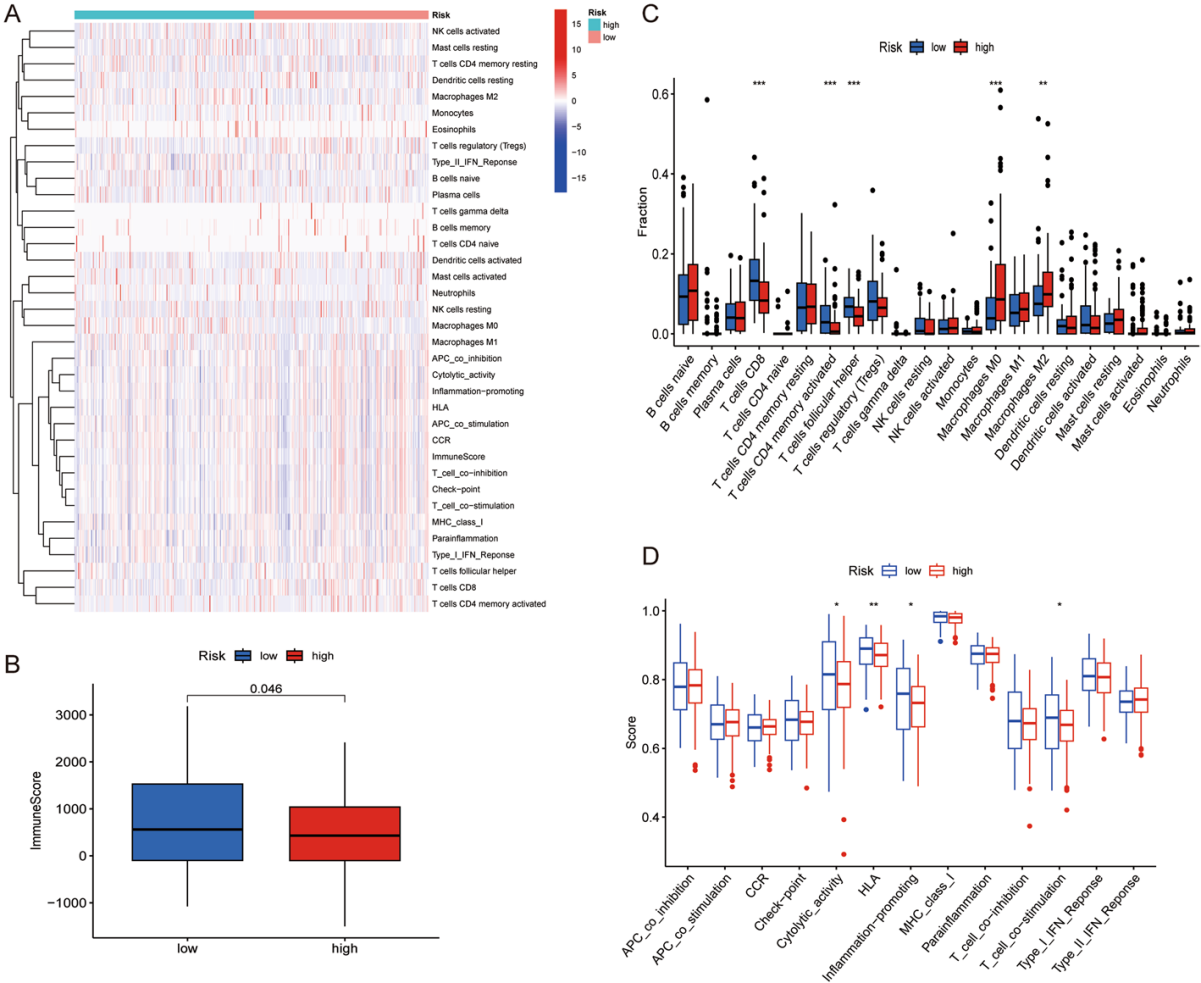
Characteristics of tumor immune microenvironment between different risk subgroups. **a** Heatmap showed the relationship between different risk subgroups and tumor immune microenvironment; **b** The comparisons of immune scores between different risk subgroups; **c** The boxplot illustrating the difference in tumor-infiltrating immune cells between different risk subgroups; **d** The boxplot illustrating the difference in immune-related functions between different risk subgroups. *P < 0.05; **P < 0.01; ***P < 0.001

We used various immune evaluation algorithms to examine the immune status in the two risk groups of BCa patients (Fig. 7a). According to the ESTIMATE results, the low- TILRGS subgroup had higher immune scores than the high-TILRGS subgroup (Fig. 7b). As illustrated in Fig. 7c. The low-TILRGS subgroup had higher proportions of CD8 T cells, memory-activated CD4 T cells, and follicular helper T cells than the high- TILRGS subgroup. While M0 and M2 macrophage fractions were higher in the high- TILRGS subgroup compared to the low-TILRGS subgroup. Additionally, we discovered that the low-TILRGS subgroup had higher scores for cytolytic activity, HLA, inflammation-promoting, and T-cell co-stimulation than the high-TILRGS subgroup (Fig. 7d).

### Immunotherapeutic responses of various risk groups

**Fig. 8:**
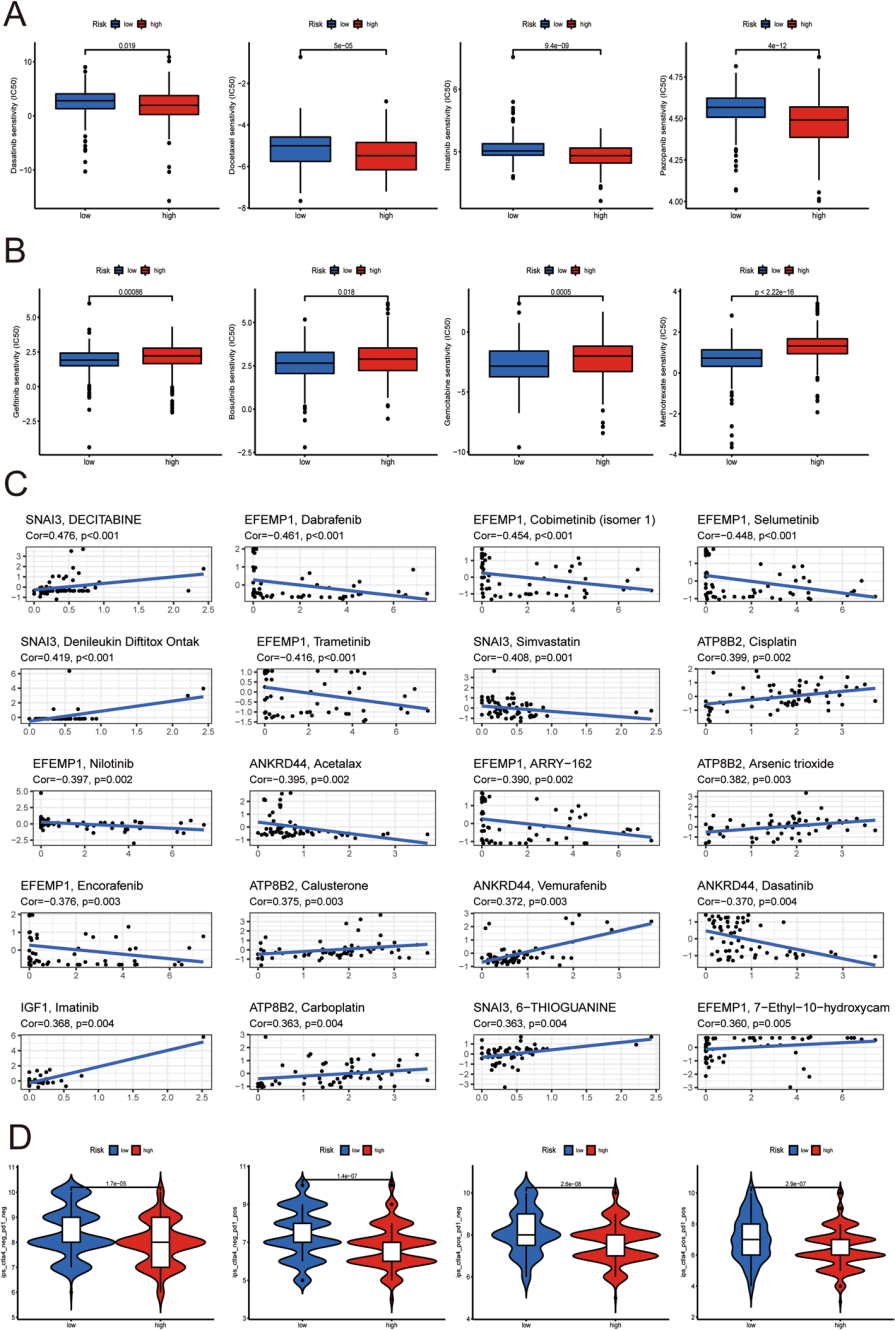
Evaluation of drug sensitivity and immunotherapy. **a**, **b** Sensitivity analysis of chemotherapeutic drugs between different risk groups; **c** The relationship between five genes in the signature and the drug sensitivity; **d** The comparison of immunophenoscore (IPS) between different risk subgroups

In the current study, we evaluated the IC50 values of several chemotherapeutic medicines concerning the various risk groups. The results showed that dasatinib, docetaxel, imatinib, and pazopanib had lower IC50 values in the high-TILRGS subgroup than in the low-TILRGS subgroup (Fig. 8a), implying that patients in the high-TILRGS subgroup can gain more from aforementioned chemotherapies, while the IC50 values of gefitinib, bosutinib, gemcitabine, and methotrexate were lower in the low-TILRGS subgroup (Fig. 8b), indicating that patients in the low-TILRGS subgroup can receive more benefit from the abovementioned chemotherapies. In this study, we aim to assess whether the hub TILRGs identified by our work are viable candidates for use as therapeutic targets. Through the CellMiner database, we examined the drug sensitivity of numerous substances that had been approved by the FDA or under clinical trials. The 20 top most significant correlations are displayed in Fig. 8c. Additionally, TCIA results showed that the low-TILRGS subgroup had higher TCIA scores compared to the high-TILRGS subgroup (Fig. 8d), indicating that these patients may profit from immunotherapy with increased anti-CTLA4 and anti-PD-1 therapeutic potency.

### Single-cell analysis of hub genes

**Fig. 9:**
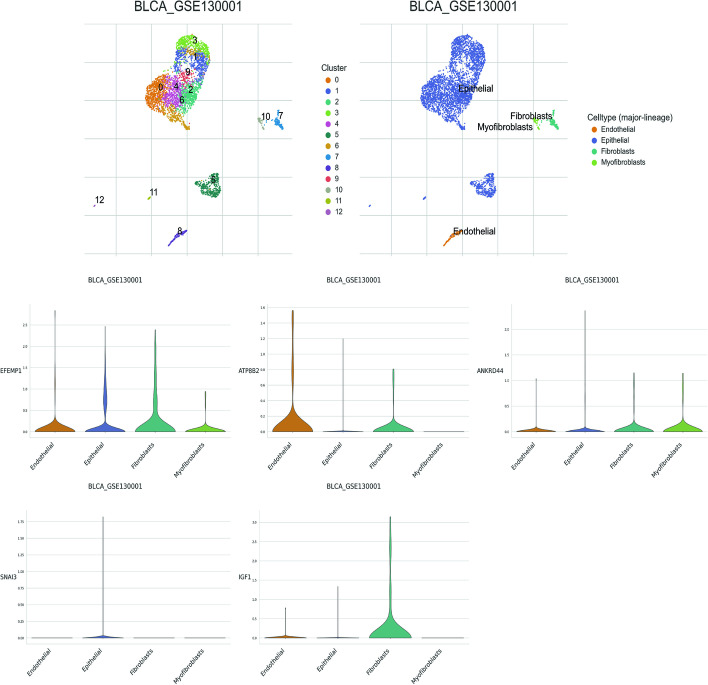
Single-cell analysis of hub genes

To investigate the expression of key genes in the TME of bladder cancer (BCa), we conducted single-cell analysis of the GSE130001 dataset obtained from the TISCH database. As depicted in Fig. 9, EFEMP1 and ATP8B2 were predominantly expressed in endothelial cells; ANKRD44 and SNAI3 were mostly observed in epithelial cells; whereas IGF1 was mainly seen in myofibroblasts.

### Identification of clusters and their relationship to clinical outcomes and tumor immunity in BCa

**Fig. 10:**
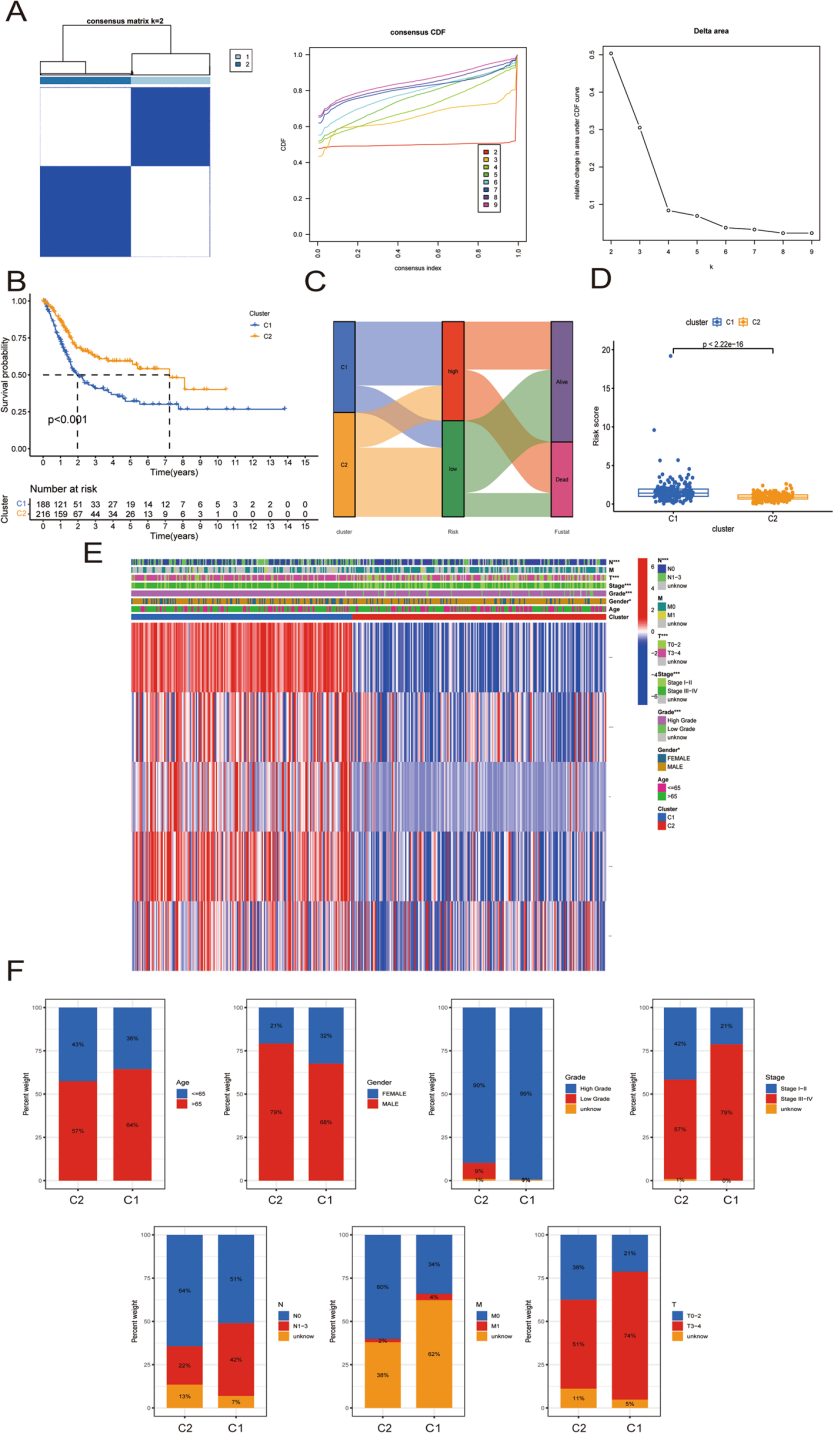
Cluster analysis of five genes in the signature in the TCGA dataset. **a** The BCa patients in the TCGA cohort was divided into two distinct clusters when k = 2; **b** The Kaplan–Meier curve survival analysis between different cluster groups; **c** The potential connection between cluster, risk score, and survival status; **d** The boxplot illustrating the difference in risk score between different clusters; **e** Heatmap showed the relationship between different clusters and clinical features; **f** Relationships between different clusters and clinical features. *P < 0.05; ***P < 0.001

**Fig. 11:**
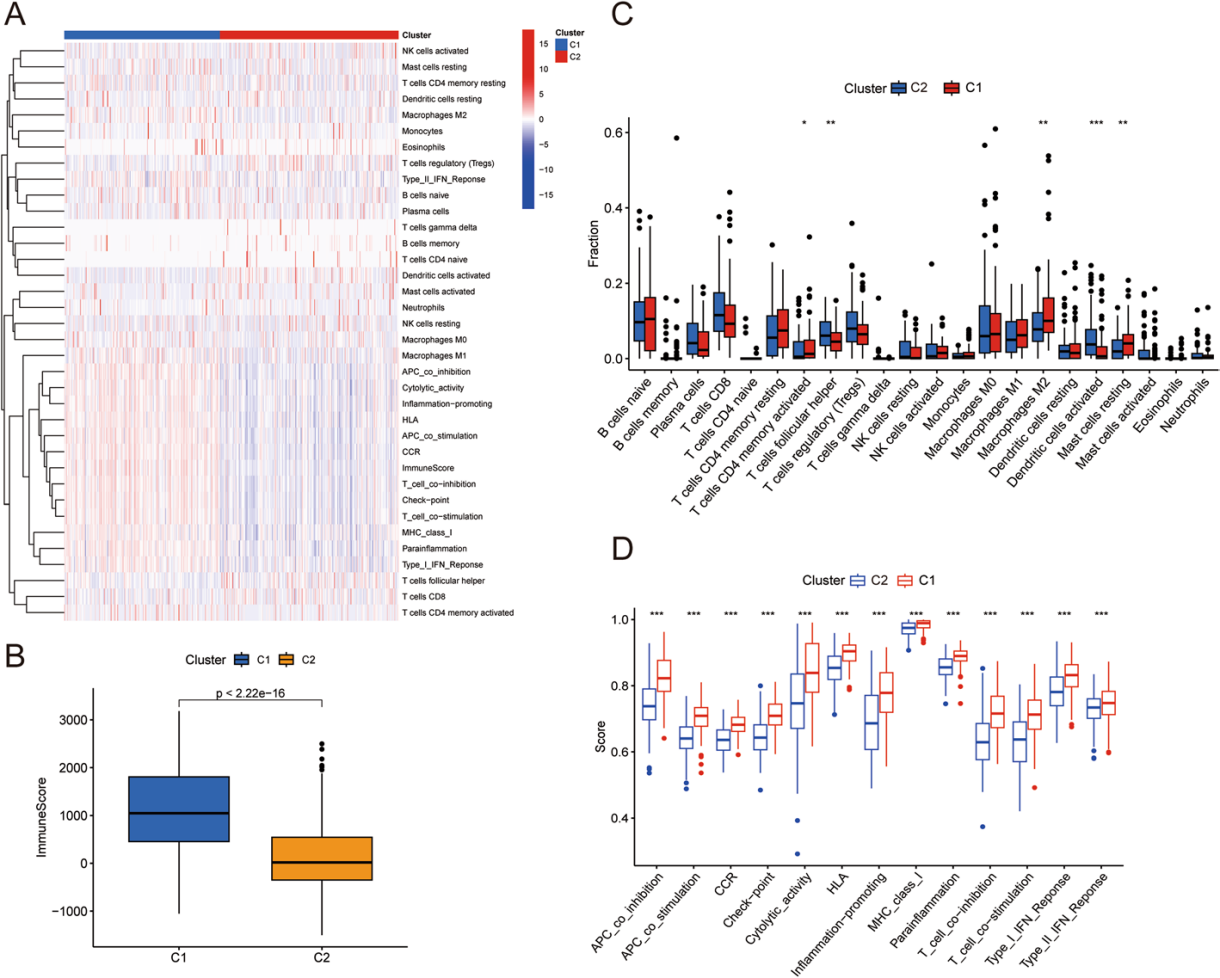
Characteristics of tumor immune microenvironment between different clusters. **a** Heatmap showed the relationship between different clusters and tumor immune microenvironment; **b** The comparisons of immune scores between different clusters; **c** The boxplot illustrating the difference in tumor-infiltrating immune cells between different clusters; **d** The boxplot illustrating the difference in immune-related functions between different clusters. *P < 0.05; **P < 0.01; ***P < 0.001

Unsupervised consensus analysis was used to separate all TCGA BCa patients into two distinct clusters (Fig. 10a). Patients in cluster 2 (n = 216) fared better than those in cluster 1 (n = 188) in terms of overall survival, according to our findings (Fig. 10b). Cluster 1 showed a fatality rate and higher risk score compared to cluster 2 (Fig. 10c, d). In the TCGA BCa dataset, we discovered substantial relationships between different clusters and gender, pathologic grade, clinical stage, T stage, and N stage (Fig. 10e). Cluster 1 had a larger proportion of patients that were female, high grade, stage III–IV, N1–3, and T3–4 than cluster 2 (Fig. 10f). Next, we examined the immune landscape in the two clusters using different immune evaluation algorithms (Fig. 11a). Cluster 1 has higher immune scores than cluster 2 based on the ESTIMATE results (Fig. 11b). The results of CIBERSORT algorithm showed that compared to cluster 2, cluster 1 exhibited enhanced levels of memory activated CD4 T cells, follicular helper T cells, M2 macrophage, activated dendritic cells, and resting mast cells (Fig. 11c). Further analysis revealed that cluster 1 outperformed cluster 2 in terms of all 13 immune-related pathways (Fig. 11d).

## Discussion

The most common cancer in the world, BCa, poses a major risk to both people's health and the global economy [25]. For NMIBC, its high rate of progression and recurrence requires ongoing invasive surveillance [2]. Additionally, the strong metastatic potential of MIBC leads to increased disease-specific mortality [3]. Numerous clinical studies supported the approval of ICIs for BCa and demonstrated the important significance of ICIs [26, 27]. However, not every patient in these trials reacted to the ICI treatment [28], highlighting the urgent need for finding biomarkers that foretell the effectiveness of ICIs. The TME is crucial to the emergence and progression of cancer. An overview of the dynamic changes in tumor-infiltrating lymphocytes make it easier to understand the potential mechanisms underlying tumor progression and to screen for novel therapeutic targets.

Herein, five TILRGs were identified in this study to create a prognostic model for BCa patients (EFEMP1, ANKRD44, IGF1, ATP8B2, and SNAI3). EFEMP1, IGF1, and SNAI3 have been linked to the development and prognosis of BCa in earlier research. ANKRD44 and ATP8B2 were rarely reported in any cancer research. For example, EFEMP1 protein expression was upregulated by METTL1-mediated m7G tRNA modification, which in turn leads to bladder cancer development [29]. Zhao et al. [30] showed that patients with BCa have higher plasma IGF1 levels than controls. Recently, the presence of EMT-related genes such as SNAI3 was linked with microbial abundance in bladder cancer tumors [31]. In comparison to the traditional clinical parameters, this gene signature shows a higher ability to predict the prognosis of BCa patients. A high-TILRGS subgroup and a low-TILRGS subgroup of BCa patients were created based on the risk scores that were generated using the prognostic model's formula. We found that a greater risk score was connected to a higher degree of clinical features, signifying a worsened prognosis for BC patients. Furthermore, even after accounting for other clinical factors, the gene signature was confirmed to be a reliable prognostic factor. Finally, a predictive nomogram that included age and risk score was created and its applicability was evaluated. All findings indicated that the designed nomogram had a reasonable level of prediction discrimination in monitoring BC patients' OS.

The low-TILRGS subgroup had a higher immune score, higher levels of immune cell infiltration, and higher levels of immune pathway activation, while the levels of M2 macrophage infiltration was greater in the high-TILRGS subgroup, indicating a substantial difference in immune status across the various groups and immunosuppression may play a role in the high-TILRGS subgroup. Moreover, according to the immunotherapy response results, patients in the low-TILRGS subgroup responded better to ICIs.

TME plays a pivotal role in the growth, migration, invasion and metastasis of cancer cells and may influence tumor recurrence and progression. An interesting finding of this study was that the immune landscape was significantly different between the various groups. Overall, the low-TILRGS subgroup showed elevated immune scores, higher infiltration of CD8 T cells, memory-activated CD4 T cells and follicular helper T cells, as well as increased activation of immune pathways. In contrast, high levels of infiltration of M0 and M2 macrophages were observed in the high-TILRGS subgroup. Of these, M0 macrophages are dormant macrophages that can be pushed to differentiate into M1 and M2 macrophages. In addition, M2 macrophages possess immunosuppressive capacities when activated, secreting certain immunosuppressive factors that can inhibit T-cell activation and thus suppress immune functions. Research has demonstrated that M2 macrophages are the most prevalent type of bladder cancer-associated macrophages that promote bladder cancer development; hence, we attempted to connect the immunosuppression caused by M2 macrophages to the lowered overall survival rate observed in the high-TILRGS subgroup.

The current study contains several drawbacks. First, using retroactive data from the TCGA and GEO databases, the gene signature was built and verified. To assess its efficacy and viability, additional large-scale prospective clinical studies are needed. Additionally, more well-planned basic research trials are required to emphasize the critical part that TILRGs play in the emergence and progression of BCa. In conclusion, the current work develops a gene signature based on TILRGs that may be used as a useful predictor of BCa patients' prognosis and response to immunotherapy.

## Supplementary Information

**Additional file 1**. **Table S1.** Identification of tumor-infiltrating lymphocyte-related genes. **Table S2.** Differentially expressed genes between the high-risk subgroup and the low-risk subgroup.

## Data Availability

The datasets used in this study can be found in the GEO database (https://www.ncbi.nlm.nih.gov/geo/), and TCGA database (https://portal.gdc.cancer.gov/).
