# Supplementary material for: Identification and validation of tumor-infiltrating lymphocyte-related prognosis signature for predicting prognosis and immunotherapeutic response in bladder cancer
Source: BMC Bioinformatics. 2023 Mar 27;24:118. doi: 10.1186/s12859-023-05241-z (PMC10041757; doi:10.1186/s12859-023-05241-z)
Supplement: Supplementary file 1 — Additional file 1. Table S1. Identification of tumor-infiltrating lymphocyte-related genes. Table S2. Differentially expressed genes between the high-risk subgroup and the low-risk subgroup. [file 12859_2023_5241_MOESM1_ESM.docx]

**TableS1.** Identification of tumor-infiltrating lymphocyte-related genes.

| Cell | Gene | Cor | P-value |
| --- | --- | --- | --- |
| TIL | SASH3 | 0.965 | 1.67E-235 |
| TIL | EVI2B | 0.959 | 5.38E-223 |
| TIL | SLAMF6 | 0.958 | 7.05E-221 |
| TIL | SLAMF1 | 0.956 | 5.18E-216 |
| TIL | CD3E | 0.954 | 2.41E-213 |
| TIL | P2RY10 | 0.951 | 9.71E-208 |
| TIL | CD48 | 0.949 | 6.69E-204 |
| TIL | TRAC | 0.948 | 0.00E+00 |
| TIL | CD2 | 0.947 | 2.82E-200 |
| TIL | RHOH | 0.945 | 5.89E-198 |
| TIL | PTPRC | 0.945 | 1.57E-196 |
| TIL | ARHGAP9 | 0.941 | 2.31E-191 |
| TIL | IL10RA | 0.940 | 4.99E-190 |
| TIL | IL21R | 0.937 | 4.48E-185 |
| TIL | SELPLG | 0.936 | 0.00E+00 |
| TIL | PTPN7 | 0.936 | 3.34E-184 |
| TIL | CD53 | 0.935 | 0.00E+00 |
| TIL | SH2D1A | 0.934 | 5.20E-182 |
| TIL | ITK | 0.934 | 2.42E-181 |
| TIL | NCKAP1L | 0.931 | 0.00E+00 |
| TIL | SNX20 | 0.931 | 1.92E-177 |
| TIL | SPN | 0.930 | 2.24E-177 |
| TIL | CD52 | 0.930 | 0.00E+00 |
| TIL | CYTIP | 0.930 | 0.00E+00 |
| TIL | WAS | 0.929 | 0.00E+00 |
| TIL | TRBC1 | 0.927 | 0.00E+00 |
| TIL | CXCR3 | 0.925 | 5.49E-171 |
| TIL | LCP2 | 0.923 | 8.43E-169 |
| TIL | ITGAL | 0.922 | 1.01E-167 |
| TIL | PLEK | 0.922 | 0.00E+00 |
| TIL | CCR5 | 0.922 | 1.82E-167 |
| TIL | RASAL3 | 0.921 | 1.28E-166 |
| TIL | BTK | 0.921 | 3.50E-166 |
| TIL | SLA | 0.920 | 7.26E-166 |
| TIL | MYO1G | 0.920 | 3.92E-165 |
| TIL | GPR65 | 0.919 | 1.25E-164 |
| TIL | CYTH4 | 0.918 | 1.99E-163 |
| TIL | GPR174 | 0.918 | 2.10E-163 |
| TIL | BIN2 | 0.918 | 2.10E-163 |
| TIL | PYHIN1 | 0.917 | 1.37E-162 |
| TIL | DOK2 | 0.914 | 2.05E-159 |
| TIL | DOCK2 | 0.914 | 3.18E-159 |
| TIL | AOAH | 0.914 | 3.74E-159 |
| TIL | UBASH3A | 0.913 | 1.31E-158 |
| TIL | MPEG1 | 0.912 | 4.20E-158 |
| TIL | NCF1 | 0.912 | 1.93E-157 |
| TIL | SLA2 | 0.909 | 3.67E-155 |
| TIL | CD37 | 0.909 | 0.00E+00 |
| TIL | SIT1 | 0.908 | 5.88E-154 |
| TIL | TBC1D10C | 0.908 | 1.27E-153 |
| TIL | CXorf21 | 0.907 | 5.85E-153 |
| TIL | WDFY4 | 0.907 | 1.09E-152 |
| TIL | SPI1 | 0.906 | 0.00E+00 |
| TIL | CD84 | 0.904 | 4.11E-150 |
| TIL | SCIMP | 0.903 | 3.43E-149 |
| TIL | IL12RB1 | 0.902 | 7.45E-149 |
| TIL | PCED1B-AS1 | 0.902 | 7.92E-149 |
| TIL | ITGB2 | 0.902 | 1.58E-148 |
| TIL | CYBB | 0.901 | 0.00E+00 |
| TIL | MS4A6A | 0.899 | 3.24E-146 |
| TIL | PDCD1 | 0.899 | 3.64E-146 |
| TIL | CST7 | 0.897 | 7.43E-145 |
| TIL | MYO1F | 0.897 | 0.00E+00 |
| TIL | AIF1 | 0.897 | 0.00E+00 |
| TIL | SAMSN1 | 0.897 | 3.07E-144 |
| TIL | GRAP2 | 0.896 | 3.35E-144 |
| TIL | EVI2A | 0.896 | 8.82E-144 |
| TIL | ZNF831 | 0.896 | 1.68E-143 |
| TIL | IRF4 | 0.895 | 3.88E-143 |
| TIL | CD5 | 0.894 | 1.49E-142 |
| TIL | BTLA | 0.894 | 2.39E-142 |
| TIL | IL2RA | 0.894 | 3.54E-142 |
| TIL | TNFAIP8L2 | 0.894 | 7.38E-142 |
| TIL | GPR171 | 0.893 | 1.20E-141 |
| TIL | IKZF1 | 0.892 | 5.17E-141 |
| TIL | LAIR1 | 0.891 | 5.32E-140 |
| TIL | IRF8 | 0.890 | 1.84E-139 |
| TIL | HAVCR2 | 0.890 | 0.00E+00 |
| TIL | MNDA | 0.890 | 5.72E-139 |
| TIL | C1QA | 0.889 | 1.47E-138 |
| TIL | GZMK | 0.889 | 1.52E-138 |
| TIL | PARVG | 0.889 | 2.38E-138 |
| TIL | PIK3R5 | 0.888 | 5.20E-138 |
| TIL | TRBV20-1 | 0.888 | 1.75E-137 |
| TIL | PTPN22 | 0.887 | 6.68E-137 |
| TIL | NKG7 | 0.886 | 0.00E+00 |
| TIL | FERMT3 | 0.886 | 0.00E+00 |
| TIL | NCF1C | 0.886 | 5.25E-136 |
| TIL | LY9 | 0.886 | 5.64E-136 |
| TIL | LST1 | 0.885 | 6.90E-136 |
| TIL | TRG-AS1 | 0.885 | 1.00E-135 |
| TIL | ICOS | 0.885 | 1.16E-135 |
| TIL | LILRB4 | 0.885 | 1.52E-135 |
| TIL | NCF1B | 0.884 | 5.24E-135 |
| TIL | LILRB2 | 0.884 | 6.99E-135 |
| TIL | TRAT1 | 0.884 | 9.00E-135 |
| TIL | CD27 | 0.883 | 2.67E-134 |
| TIL | TLR8 | 0.882 | 9.04E-134 |
| TIL | CMKLR1 | 0.882 | 2.15E-133 |
| TIL | TYROBP | 0.881 | 0.00E+00 |
| TIL | CD3G | 0.881 | 8.58E-133 |
| TIL | LILRB1 | 0.881 | 9.85E-133 |
| TIL | C1QB | 0.880 | 0.00E+00 |
| TIL | LINC00426 | 0.879 | 1.70E-131 |
| TIL | C1QC | 0.878 | 0.00E+00 |
| TIL | SLAMF8 | 0.877 | 3.44E-130 |
| TIL | CD3D | 0.877 | 4.24E-130 |
| TIL | TNFSF13B | 0.877 | 5.74E-130 |
| TIL | CTLA4 | 0.877 | 1.02E-129 |
| TIL | SIGLEC10 | 0.876 | 1.28E-129 |
| TIL | IL16 | 0.876 | 1.99E-129 |
| TIL | CD28 | 0.876 | 2.49E-129 |
| TIL | HLA-DQA1 | 0.876 | 0.00E+00 |
| TIL | AC243960.1 | 0.875 | 5.17E-129 |
| TIL | RNASE6 | 0.875 | 1.49E-128 |
| TIL | JAML | 0.874 | 3.73E-128 |
| TIL | TAGAP | 0.874 | 4.94E-128 |
| TIL | P2RY13 | 0.873 | 2.66E-127 |
| TIL | FGL2 | 0.872 | 0.00E+00 |
| TIL | LAT2 | 0.872 | 1.03E-126 |
| TIL | TAF5LP1 | 0.872 | 1.75E-126 |
| TIL | C3AR1 | 0.871 | 5.38E-126 |
| TIL | SP140 | 0.870 | 9.54E-126 |
| TIL | HLA-DPB1 | 0.870 | 0.00E+00 |
| TIL | CD7 | 0.870 | 0.00E+00 |
| TIL | FCER1G | 0.868 | 1.90E-124 |
| TIL | C1orf162 | 0.867 | 5.62E-124 |
| TIL | TIGIT | 0.867 | 8.65E-124 |
| TIL | TRBV7-9 | 0.867 | 1.82E-123 |
| TIL | IGSF6 | 0.866 | 4.32E-123 |
| TIL | ABI3 | 0.866 | 7.14E-123 |
| TIL | CD300LF | 0.865 | 1.47E-122 |
| TIL | TRBV5-1 | 0.864 | 6.91E-122 |
| TIL | CD247 | 0.864 | 1.41E-121 |
| TIL | TBX21 | 0.862 | 9.52E-121 |
| TIL | IL2RG | 0.861 | 5.59E-120 |
| TIL | GVINP1 | 0.861 | 7.39E-120 |
| TIL | AC004847.1 | 0.860 | 1.43E-119 |
| TIL | HCK | 0.860 | 1.91E-119 |
| TIL | NFAM1 | 0.859 | 7.05E-119 |
| TIL | CD4 | 0.859 | 0.00E+00 |
| TIL | AC090559.1 | 0.859 | 1.06E-118 |
| TIL | PRF1 | 0.859 | 1.12E-118 |
| TIL | CD226 | 0.858 | 1.97E-118 |
| TIL | GZMA | 0.858 | 2.50E-118 |
| TIL | LAX1 | 0.858 | 2.74E-118 |
| TIL | GIMAP4 | 0.858 | 0.00E+00 |
| TIL | LY86 | 0.857 | 5.73E-118 |
| TIL | THEMIS | 0.857 | 6.69E-118 |
| TIL | FCRL3 | 0.856 | 2.75E-117 |
| TIL | ZAP70 | 0.855 | 6.95E-117 |
| TIL | SCML4 | 0.855 | 9.56E-117 |
| TIL | CD86 | 0.854 | 4.13E-116 |
| TIL | CCR2 | 0.854 | 7.04E-116 |
| TIL | HLA-DMB | 0.853 | 1.37E-115 |
| TIL | ICAM3 | 0.853 | 2.53E-115 |
| TIL | LTA | 0.852 | 2.80E-115 |
| TIL | TRBV7-2 | 0.851 | 1.20E-114 |
| TIL | IL2RB | 0.850 | 3.94E-114 |
| TIL | LINC00996 | 0.850 | 6.36E-114 |
| TIL | CALHM6 | 0.849 | 1.40E-113 |
| TIL | SRGN | 0.849 | 0.00E+00 |
| TIL | CEACAM21 | 0.849 | 2.80E-113 |
| TIL | ZC3H12D | 0.848 | 4.28E-113 |
| TIL | CCL4 | 0.848 | 6.05E-113 |
| TIL | LRRC25 | 0.848 | 1.07E-112 |
| TIL | NLRC3 | 0.847 | 1.77E-112 |
| TIL | SIGLEC1 | 0.847 | 3.14E-112 |
| TIL | TNFSF8 | 0.846 | 5.77E-112 |
| TIL | FUT7 | 0.845 | 2.32E-111 |
| TIL | AL590764.1 | 0.845 | 3.95E-111 |
| TIL | CD180 | 0.844 | 6.01E-111 |
| TIL | RGS18 | 0.844 | 8.05E-111 |
| TIL | GPR183 | 0.844 | 9.16E-111 |
| TIL | TNFRSF9 | 0.843 | 1.82E-110 |
| TIL | TIFAB | 0.843 | 2.81E-110 |
| TIL | CD6 | 0.842 | 8.14E-110 |
| TIL | TRBV19 | 0.842 | 1.21E-109 |
| TIL | CXCR6 | 0.842 | 1.23E-109 |
| TIL | GAB3 | 0.841 | 1.91E-109 |
| TIL | FAM78A | 0.841 | 1.98E-109 |
| TIL | LTB | 0.841 | 0.00E+00 |
| TIL | WIPF1 | 0.840 | 0.00E+00 |
| TIL | SIRPG | 0.839 | 1.89E-108 |
| TIL | PIK3AP1 | 0.839 | 2.56E-108 |
| TIL | CD33 | 0.839 | 4.12E-108 |
| TIL | SAMD3 | 0.839 | 4.43E-108 |
| TIL | CD8A | 0.838 | 8.00E-108 |
| TIL | BCL2A1 | 0.837 | 2.43E-107 |
| TIL | CLEC10A | 0.836 | 5.27E-107 |
| TIL | ACAP1 | 0.836 | 5.59E-107 |
| TIL | HLA-DRA | 0.836 | 0.00E+00 |
| TIL | MS4A4A | 0.836 | 0.00E+00 |
| TIL | CRTAM | 0.835 | 1.62E-106 |
| TIL | GBP5 | 0.834 | 4.46E-106 |
| TIL | CSF1R | 0.834 | 0.00E+00 |
| TIL | TMEM150B | 0.832 | 7.63E-105 |
| TIL | GZMB | 0.831 | 2.70E-104 |
| TIL | TRAV4 | 0.830 | 4.01E-104 |
| TIL | GZMH | 0.830 | 9.17E-104 |
| TIL | FOXP3 | 0.829 | 2.47E-103 |
| TIL | TRBV29-1 | 0.829 | 2.68E-103 |
| TIL | DOCK10 | 0.828 | 4.98E-103 |
| TIL | SLCO2B1 | 0.827 | 1.20E-102 |
| TIL | CD80 | 0.827 | 1.44E-102 |
| TIL | GGTA1 | 0.826 | 2.37E-102 |
| TIL | FCGR1A | 0.825 | 6.82E-102 |
| TIL | TRAF3IP3 | 0.825 | 8.58E-102 |
| TIL | KLRB1 | 0.825 | 9.55E-102 |
| TIL | TRGC2 | 0.825 | 1.27E-101 |
| TIL | GNGT2 | 0.824 | 1.99E-101 |
| TIL | RCSD1 | 0.824 | 2.80E-101 |
| TIL | CXCL9 | 0.824 | 0.00E+00 |
| TIL | SLC7A7 | 0.824 | 5.24E-101 |
| TIL | IL7R | 0.823 | 6.02E-101 |
| TIL | PDCD1LG2 | 0.823 | 9.41E-101 |
| TIL | HNRNPA1P21 | 0.822 | 1.66E-100 |
| TIL | CLEC4E | 0.822 | 2.19E-100 |
| TIL | GZMM | 0.821 | 5.62E-100 |
| TIL | ITGAX | 0.821 | 0.00E+00 |
| TIL | FCGR3A | 0.821 | 0.00E+00 |
| TIL | CXCL13 | 0.820 | 1.18E-99 |
| TIL | CD163 | 0.819 | 3.87E-99 |
| TIL | CD14 | 0.819 | 0.00E+00 |
| TIL | CD74 | 0.819 | 0.00E+00 |
| TIL | CORO1A | 0.819 | 7.16E-99 |
| TIL | LCK | 0.818 | 8.81E-99 |
| TIL | TFEC | 0.818 | 1.08E-98 |
| TIL | VSIG4 | 0.817 | 4.04E-98 |
| TIL | LAPTM5 | 0.817 | 4.05E-98 |
| TIL | LILRB3 | 0.817 | 4.39E-98 |
| TIL | FTH1P22 | 0.817 | 5.14E-98 |
| TIL | HK3 | 0.816 | 9.14E-98 |
| TIL | HLA-DPA1 | 0.816 | 0.00E+00 |
| TIL | TRAV17 | 0.816 | 1.13E-97 |
| TIL | ABCD2 | 0.815 | 2.77E-97 |
| TIL | CEACAM4 | 0.813 | 1.14E-96 |
| TIL | SIGLEC9 | 0.813 | 1.44E-96 |
| TIL | SIGLEC7 | 0.813 | 2.14E-96 |
| TIL | LINC01934 | 0.813 | 2.21E-96 |
| TIL | KLHL6 | 0.813 | 2.69E-96 |
| TIL | FPR3 | 0.812 | 4.17E-96 |
| TIL | ALOX5AP | 0.811 | 0.00E+00 |
| TIL | GIMAP1 | 0.811 | 1.38E-95 |
| TIL | KCNA3 | 0.809 | 5.74E-95 |
| TIL | AL365361.1 | 0.809 | 5.88E-95 |
| TIL | NCR3 | 0.809 | 8.29E-95 |
| TIL | CCL5 | 0.809 | 0.00E+00 |
| TIL | TRAV13-1 | 0.808 | 1.70E-94 |
| TIL | LINC01871 | 0.808 | 2.51E-94 |
| TIL | ARHGAP15 | 0.807 | 3.76E-94 |
| TIL | MFNG | 0.807 | 3.97E-94 |
| TIL | RASGRP4 | 0.807 | 5.37E-94 |
| TIL | MAP4K1 | 0.806 | 1.48E-93 |
| TIL | FCGR2A | 0.805 | 2.21E-93 |
| TIL | CCR4 | 0.805 | 2.90E-93 |
| TIL | CD38 | 0.805 | 3.47E-93 |
| TIL | HLA-DRB1 | 0.805 | 0.00E+00 |
| TIL | CD209 | 0.804 | 7.53E-93 |
| TIL | PILRA | 0.804 | 9.42E-93 |
| TIL | STAT4 | 0.804 | 1.33E-92 |
| TIL | PLA2G2D | 0.803 | 1.57E-92 |
| TIL | PIK3R6 | 0.803 | 1.63E-92 |
| TIL | TMEM273 | 0.803 | 2.17E-92 |
| TIL | ADGRG5 | 0.802 | 3.59E-92 |
| TIL | C11orf21 | 0.802 | 4.07E-92 |
| TIL | PPP1R16B | 0.802 | 4.28E-92 |
| TIL | CD300A | 0.802 | 4.94E-92 |
| TIL | CD300C | 0.802 | 5.33E-92 |
| TIL | NAPSB | 0.802 | 7.71E-92 |
| TIL | ZBP1 | 0.802 | 8.24E-92 |
| TIL | LAG3 | 0.801 | 1.42E-91 |
| TIL | TBXAS1 | 0.799 | 5.46E-91 |
| TIL | ITGAM | 0.799 | 7.80E-91 |
| TIL | RGS1 | 0.799 | 0.00E+00 |
| TIL | HCST | 0.797 | 5.44E-90 |
| TIL | MRC1 | 0.797 | 6.82E-90 |
| TIL | TRBV27 | 0.796 | 7.51E-90 |
| TIL | NLRP3 | 0.796 | 8.54E-90 |
| TIL | LILRA1 | 0.796 | 1.56E-89 |
| TIL | PSTPIP1 | 0.795 | 2.00E-89 |
| TIL | CSF2RB | 0.794 | 7.57E-89 |
| TIL | TRAV12-3 | 0.794 | 8.72E-89 |
| TIL | FCGR1B | 0.792 | 4.14E-88 |
| TIL | GIMAP7 | 0.791 | 0.00E+00 |
| TIL | ADAMDEC1 | 0.791 | 7.78E-88 |
| TIL | TRAV8-6 | 0.790 | 1.46E-87 |
| TIL | HLA-DQB1 | 0.790 | 0.00E+00 |
| TIL | CLECL1 | 0.789 | 3.76E-87 |
| TIL | TRAV12-2 | 0.789 | 4.21E-87 |
| TIL | LYZ | 0.789 | 0.00E+00 |
| TIL | PRKAR1B-AS1 | 0.789 | 6.14E-87 |
| TIL | TESPA1 | 0.788 | 7.10E-87 |
| TIL | LINC01943 | 0.788 | 7.99E-87 |
| TIL | CELF2 | 0.787 | 3.31E-86 |
| TIL | GPR34 | 0.786 | 5.38E-86 |
| TIL | LY96 | 0.786 | 5.51E-86 |
| TIL | MZB1 | 0.786 | 5.52E-86 |
| TIL | EBI3 | 0.786 | 6.51E-86 |
| TIL | TRBV9 | 0.786 | 6.57E-86 |
| TIL | PDE4B | 0.786 | 7.36E-86 |
| TIL | LINC01358 | 0.785 | 8.89E-86 |
| TIL | RASSF4 | 0.785 | 1.54E-85 |
| TIL | IL18RAP | 0.784 | 4.25E-85 |
| TIL | IFFO1 | 0.783 | 6.55E-85 |
| TIL | AC015911.3 | 0.783 | 6.57E-85 |
| TIL | CD40LG | 0.783 | 9.07E-85 |
| TIL | MIR155HG | 0.782 | 1.36E-84 |
| TIL | RUNX3 | 0.782 | 1.87E-84 |
| TIL | LILRB5 | 0.782 | 2.02E-84 |
| TIL | LINC01281 | 0.781 | 2.83E-84 |
| TIL | LILRA6 | 0.781 | 3.34E-84 |
| TIL | STAP1 | 0.781 | 3.45E-84 |
| TIL | IL4I1 | 0.781 | 0.00E+00 |
| TIL | LINC02446 | 0.780 | 6.79E-84 |
| TIL | FLI1 | 0.780 | 7.42E-84 |
| TIL | AC104530.1 | 0.780 | 8.37E-84 |
| TIL | IGHM | 0.779 | 1.59E-83 |
| TIL | FASLG | 0.779 | 1.88E-83 |
| TIL | KLRD1 | 0.778 | 2.52E-83 |
| TIL | ITGB2-AS1 | 0.778 | 3.41E-83 |
| TIL | AC004921.1 | 0.778 | 4.90E-83 |
| TIL | TOMM20P2 | 0.778 | 4.97E-83 |
| TIL | LILRA5 | 0.777 | 6.05E-83 |
| TIL | SPOCK2 | 0.777 | 8.92E-83 |
| TIL | CD79A | 0.776 | 1.41E-82 |
| TIL | FMNL1 | 0.776 | 1.66E-82 |
| TIL | TRBV18 | 0.776 | 2.07E-82 |
| TIL | TRAV2 | 0.776 | 2.40E-82 |
| TIL | TRAV8-4 | 0.775 | 3.14E-82 |
| TIL | TRAV26-1 | 0.775 | 4.34E-82 |
| TIL | FCRL6 | 0.774 | 1.10E-81 |
| TIL | FPR1 | 0.774 | 1.16E-81 |
| TIL | PEAK3 | 0.773 | 1.87E-81 |
| TIL | CXCR2P1 | 0.772 | 3.59E-81 |
| TIL | TRAV12-1 | 0.770 | 2.11E-80 |
| TIL | CASP4LP | 0.769 | 3.10E-80 |
| TIL | OSCAR | 0.769 | 0.00E+00 |
| TIL | LINC00861 | 0.769 | 4.89E-80 |
| TIL | RGL4 | 0.769 | 5.67E-80 |
| TIL | TMEM176B | 0.768 | 0.00E+00 |
| TIL | AC006033.2 | 0.768 | 8.03E-80 |
| TIL | TLR10 | 0.767 | 1.46E-79 |
| TIL | CCL3 | 0.767 | 2.03E-79 |
| TIL | FYB1 | 0.767 | 2.31E-79 |
| TIL | HLA-DRB5 | 0.766 | 0.00E+00 |
| TIL | TRBV6-5 | 0.765 | 6.18E-79 |
| TIL | NCF2 | 0.765 | 7.21E-79 |
| TIL | ARHGAP25 | 0.765 | 7.44E-79 |
| TIL | FLT3 | 0.765 | 1.09E-78 |
| TIL | THEMIS2 | 0.764 | 0.00E+00 |
| TIL | LILRA2 | 0.763 | 3.93E-78 |
| TIL | LINC01857 | 0.763 | 4.41E-78 |
| TIL | TRAV3 | 0.762 | 5.54E-78 |
| TIL | TRBV5-4 | 0.762 | 6.72E-78 |
| TIL | TRAV9-2 | 0.762 | 9.28E-78 |
| TIL | TRBV2 | 0.761 | 1.09E-77 |
| TIL | MSR1 | 0.760 | 2.45E-77 |
| TIL | TSPAN32 | 0.760 | 2.65E-77 |
| TIL | IGHG1 | 0.760 | 0.00E+00 |
| TIL | STAB1 | 0.760 | 0.00E+00 |
| TIL | SSTR3 | 0.760 | 4.07E-77 |
| TIL | IGHG3 | 0.758 | 0.00E+00 |
| TIL | TRAV8-3 | 0.758 | 1.25E-76 |
| TIL | IGLC3 | 0.758 | 1.80E-76 |
| TIL | S1PR4 | 0.757 | 2.08E-76 |
| TIL | TNIP3 | 0.756 | 6.06E-76 |
| TIL | TRPV2 | 0.756 | 6.43E-76 |
| TIL | SLC15A3 | 0.756 | 7.47E-76 |
| TIL | TMC8 | 0.755 | 0.00E+00 |
| TIL | FOLR2 | 0.755 | 1.26E-75 |
| TIL | LINC01094 | 0.754 | 1.98E-75 |
| TIL | TRBV3-1 | 0.754 | 2.10E-75 |
| TIL | TRBV4-1 | 0.754 | 2.58E-75 |
| TIL | TRAV21 | 0.754 | 2.76E-75 |
| TIL | JAK3 | 0.753 | 3.48E-75 |
| TIL | PLXNC1 | 0.753 | 4.18E-75 |
| TIL | IL21 | 0.753 | 5.01E-75 |
| TIL | RUBCNL | 0.753 | 6.06E-75 |
| TIL | SLAMF7 | 0.752 | 0.00E+00 |
| TIL | CCL19 | 0.752 | 1.22E-74 |
| TIL | LINC02325 | 0.751 | 1.52E-74 |
| TIL | IL32 | 0.751 | 0.00E+00 |
| TIL | ITGA4 | 0.751 | 1.96E-74 |
| TIL | IGHV3-21 | 0.750 | 3.82E-74 |
| TIL | FCRL5 | 0.750 | 4.83E-74 |
| TIL | CCL8 | 0.749 | 5.52E-74 |
| TIL | IGKC | 0.749 | 0.00E+00 |
| TIL | VNN2 | 0.749 | 9.52E-74 |
| TIL | TRBV12-4 | 0.748 | 1.18E-73 |
| TIL | TRBV4-2 | 0.748 | 1.18E-73 |
| TIL | TNFRSF13B | 0.748 | 1.19E-73 |
| TIL | FCGR2B | 0.748 | 1.24E-73 |
| TIL | TRBV11-2 | 0.748 | 1.70E-73 |
| TIL | LINC02195 | 0.747 | 2.83E-73 |
| TIL | IGLC2 | 0.747 | 0.00E+00 |
| TIL | TRGC1 | 0.747 | 3.96E-73 |
| TIL | CCR8 | 0.746 | 4.86E-73 |
| TIL | EOMES | 0.746 | 5.05E-73 |
| TIL | TNFRSF4 | 0.746 | 6.93E-73 |
| TIL | IGKV4-1 | 0.746 | 7.16E-73 |
| TIL | IGKV3-11 | 0.745 | 8.96E-73 |
| TIL | IGHV3-23 | 0.745 | 1.28E-72 |
| TIL | TRAV16 | 0.744 | 2.06E-72 |
| TIL | GYPC | 0.744 | 2.11E-72 |
| TIL | GPR141 | 0.744 | 2.51E-72 |
| TIL | IGKV1-5 | 0.743 | 3.75E-72 |
| TIL | LGALS2 | 0.743 | 5.14E-72 |
| TIL | C1R | 0.742 | 0.00E+00 |
| TIL | BATF3 | 0.742 | 9.12E-72 |
| TIL | CIITA | 0.742 | 1.07E-71 |
| TIL | TRAV38-2DV8 | 0.741 | 1.90E-71 |
| TIL | TRAV8-2 | 0.740 | 2.47E-71 |
| TIL | CCL18 | 0.740 | 2.72E-71 |
| TIL | ATP8B4 | 0.740 | 2.91E-71 |
| TIL | IGKV3-20 | 0.739 | 4.53E-71 |
| TIL | TNFRSF1B | 0.739 | 0.00E+00 |
| TIL | CASS4 | 0.739 | 7.55E-71 |
| TIL | AC004687.1 | 0.738 | 1.08E-70 |
| TIL | C5AR1 | 0.738 | 1.15E-70 |
| TIL | IGLV1-40 | 0.738 | 1.27E-70 |
| TIL | CHRM3-AS2 | 0.738 | 1.49E-70 |
| TIL | PTGDR | 0.737 | 2.22E-70 |
| TIL | CR1 | 0.737 | 2.34E-70 |
| TIL | BFSP2 | 0.737 | 2.39E-70 |
| TIL | HAPLN3 | 0.737 | 0.00E+00 |
| TIL | SERPING1 | 0.737 | 0.00E+00 |
| TIL | PLEKHO2 | 0.736 | 3.15E-70 |
| TIL | CCR1 | 0.736 | 3.22E-70 |
| TIL | CXCR4 | 0.736 | 0.00E+00 |
| TIL | IFNG | 0.736 | 4.24E-70 |
| TIL | TNFSF14 | 0.736 | 4.68E-70 |
| TIL | TRBV5-6 | 0.736 | 4.73E-70 |
| TIL | AL133371.2 | 0.736 | 5.57E-70 |
| TIL | IGHV3-33 | 0.735 | 7.32E-70 |
| TIL | TRBV28 | 0.735 | 8.36E-70 |
| TIL | HLA-DOA | 0.735 | 0.00E+00 |
| TIL | HLA-DRB6 | 0.734 | 0.00E+00 |
| TIL | FCGR1CP | 0.734 | 1.78E-69 |
| TIL | TNFRSF17 | 0.734 | 2.07E-69 |
| TIL | IGHV3-30 | 0.733 | 2.26E-69 |
| TIL | GNLY | 0.732 | 0.00E+00 |
| TIL | CCL4L2 | 0.732 | 5.29E-69 |
| TIL | IGHV4-34 | 0.732 | 5.62E-69 |
| TIL | CXCL10 | 0.732 | 0.00E+00 |
| TIL | CD300LB | 0.731 | 8.15E-69 |
| TIL | GPRIN3 | 0.731 | 8.21E-69 |
| TIL | FCRL2 | 0.731 | 8.39E-69 |
| TIL | TNFRSF8 | 0.731 | 8.56E-69 |
| TIL | JCHAIN | 0.731 | 0.00E+00 |
| TIL | MEI1 | 0.731 | 1.24E-68 |
| TIL | IGLV3-1 | 0.731 | 1.38E-68 |
| TIL | C1S | 0.730 | 0.00E+00 |
| TIL | TLR1 | 0.730 | 2.09E-68 |
| TIL | PIK3CG | 0.729 | 3.08E-68 |
| TIL | TREM2 | 0.729 | 3.46E-68 |
| TIL | JAKMIP1 | 0.729 | 3.52E-68 |
| TIL | TRBV7-6 | 0.729 | 4.23E-68 |
| TIL | IGKV3-15 | 0.729 | 4.81E-68 |
| TIL | IGLV3-19 | 0.728 | 6.51E-68 |
| TIL | AL096816.1 | 0.728 | 8.39E-68 |
| TIL | IGLV2-23 | 0.728 | 8.92E-68 |
| TIL | AL021978.1 | 0.727 | 9.37E-68 |
| TIL | TRDC | 0.727 | 1.09E-67 |
| TIL | IGLV1-44 | 0.726 | 2.61E-67 |
| TIL | PIK3CD | 0.726 | 2.63E-67 |
| TIL | AL031846.1 | 0.725 | 3.33E-67 |
| TIL | IGHGP | 0.725 | 4.87E-67 |
| TIL | PRAM1 | 0.724 | 7.38E-67 |
| TIL | GPR84 | 0.724 | 8.11E-67 |
| TIL | TRGV10 | 0.724 | 9.10E-67 |
| TIL | TRBV6-2 | 0.724 | 1.00E-66 |
| TIL | GNG2 | 0.723 | 0.00E+00 |
| TIL | DOK3 | 0.723 | 1.75E-66 |
| TIL | CSF1 | 0.723 | 1.85E-66 |
| TIL | IGLV2-14 | 0.722 | 2.05E-66 |
| TIL | CD79B | 0.722 | 2.89E-66 |
| TIL | GLIPR2 | 0.721 | 0.00E+00 |
| TIL | AC006369.1 | 0.721 | 6.45E-66 |
| TIL | EMP3 | 0.720 | 0.00E+00 |
| TIL | MIAT | 0.720 | 1.11E-65 |
| TIL | AC007728.2 | 0.720 | 1.15E-65 |
| TIL | IGLV3-25 | 0.719 | 1.41E-65 |
| TIL | CD72 | 0.719 | 2.08E-65 |
| TIL | ZNF683 | 0.719 | 2.08E-65 |
| TIL | TRBD1 | 0.719 | 2.09E-65 |
| TIL | TRAV14DV4 | 0.718 | 2.43E-65 |
| TIL | PSMB9 | 0.717 | 0.00E+00 |
| TIL | OR2I1P | 0.717 | 4.69E-65 |
| TIL | ZEB2 | 0.717 | 6.10E-65 |
| TIL | CLEC4D | 0.717 | 7.23E-65 |
| TIL | ADORA3 | 0.716 | 9.04E-65 |
| TIL | LAP3 | 0.716 | 0.00E+00 |
| TIL | PTCRA | 0.716 | 1.17E-64 |
| TIL | TTC24 | 0.714 | 2.71E-64 |
| TIL | TRBV7-3 | 0.714 | 3.73E-64 |
| TIL | IGLV6-57 | 0.714 | 4.10E-64 |
| TIL | IGLV3-21 | 0.713 | 4.40E-64 |
| TIL | VIM-AS1 | 0.713 | 5.79E-64 |
| TIL | TRAV20 | 0.713 | 5.81E-64 |
| TIL | IGKJ1 | 0.713 | 6.22E-64 |
| TIL | SAMHD1 | 0.713 | 6.28E-64 |
| TIL | IGHV6-1 | 0.713 | 7.04E-64 |
| TIL | IGHV4-39 | 0.713 | 7.66E-64 |
| TIL | FAM30A | 0.712 | 9.83E-64 |
| TIL | TRAV41 | 0.712 | 1.01E-63 |
| TIL | IGLL5 | 0.712 | 1.26E-63 |
| TIL | IGHV3-15 | 0.712 | 1.30E-63 |
| TIL | GAPT | 0.712 | 1.37E-63 |
| TIL | IGHV3-49 | 0.711 | 2.36E-63 |
| TIL | NLRC4 | 0.710 | 2.88E-63 |
| TIL | IGHV3-11 | 0.710 | 3.37E-63 |
| TIL | PELATON | 0.710 | 4.00E-63 |
| TIL | VCAM1 | 0.710 | 4.41E-63 |
| TIL | TMEM176A | 0.709 | 5.95E-63 |
| TIL | IGHV1-69D | 0.709 | 6.72E-63 |
| TIL | GBP1 | 0.708 | 0.00E+00 |
| TIL | FAM20A | 0.708 | 8.98E-63 |
| TIL | AC007569.1 | 0.708 | 9.82E-63 |
| TIL | PIM2 | 0.708 | 0.00E+00 |
| TIL | CLEC9A | 0.708 | 1.08E-62 |
| TIL | NLRC5 | 0.708 | 0.00E+00 |
| TIL | CCL23 | 0.708 | 1.10E-62 |
| TIL | IGKV1-12 | 0.708 | 1.21E-62 |
| TIL | ANXA6 | 0.708 | 0.00E+00 |
| TIL | IGHV1-18 | 0.707 | 1.56E-62 |
| TIL | IGHV3-48 | 0.707 | 2.19E-62 |
| TIL | IGHV3-53 | 0.707 | 2.43E-62 |
| TIL | TRAV23DV6 | 0.706 | 3.99E-62 |
| TIL | TRBV15 | 0.705 | 4.94E-62 |
| TIL | RNASE2 | 0.705 | 5.97E-62 |
| TIL | IGHV4-59 | 0.705 | 7.13E-62 |
| TIL | CXCL11 | 0.704 | 0.00E+00 |
| TIL | IGKV1-6 | 0.704 | 8.97E-62 |
| TIL | IGHG2 | 0.704 | 0.00E+00 |
| TIL | MPP1 | 0.704 | 9.91E-62 |
| TIL | IFI30 | 0.704 | 1.32E-61 |
| TIL | APOL3 | 0.703 | 1.41E-61 |
| TIL | RTP5 | 0.703 | 1.83E-61 |
| TIL | GPR18 | 0.703 | 1.94E-61 |
| TIL | FGD2 | 0.703 | 2.12E-61 |
| TIL | PRKCB | 0.703 | 2.32E-61 |
| TIL | IDO1 | 0.702 | 2.36E-61 |
| TIL | TRAV36DV7 | 0.702 | 2.46E-61 |
| TIL | IGKV1-39 | 0.702 | 2.80E-61 |
| TIL | IL10 | 0.701 | 5.36E-61 |
| TIL | LINC02384 | 0.701 | 5.93E-61 |
| TIL | IGHV5-51 | 0.701 | 6.15E-61 |
| TIL | IGHV3-7 | 0.700 | 8.29E-61 |
| TIL | IGHV2-5 | 0.700 | 8.44E-61 |
| TIL | CD69 | 0.700 | 9.41E-61 |
| TIL | HLA-DOB | 0.700 | 9.50E-61 |
| TIL | STX11 | 0.700 | 9.82E-61 |
| TIL | TRBV10-3 | 0.700 | 9.99E-61 |
| TIL | ITM2A | 0.700 | 1.03E-60 |
| TIL | ADAP2 | 0.700 | 1.06E-60 |
| TIL | SIRPB2 | 0.700 | 1.17E-60 |
| TIL | TMIGD3 | 0.699 | 1.31E-60 |
| TIL | IGHV1-46 | 0.699 | 1.67E-60 |
| TIL | LAIR2 | 0.698 | 2.28E-60 |
| TIL | TRAV22 | 0.698 | 3.09E-60 |
| TIL | UNQ6494 | 0.698 | 3.21E-60 |
| TIL | GPR132 | 0.698 | 3.42E-60 |
| TIL | MS4A1 | 0.697 | 4.38E-60 |
| TIL | SOD2 | 0.697 | 0.00E+00 |
| TIL | IGLV2-11 | 0.697 | 5.47E-60 |
| TIL | IQGAP2 | 0.697 | 5.57E-60 |
| TIL | GIMAP5 | 0.696 | 7.43E-60 |
| TIL | CD8B | 0.696 | 8.03E-60 |
| TIL | B2M | 0.696 | 0.00E+00 |
| TIL | PLA2G7 | 0.696 | 8.89E-60 |
| TIL | BLK | 0.696 | 1.03E-59 |
| TIL | CASP5 | 0.695 | 1.15E-59 |
| TIL | IGKV2-30 | 0.695 | 1.20E-59 |
| TIL | IGHV4-31 | 0.695 | 1.22E-59 |
| TIL | LINC02611 | 0.695 | 1.42E-59 |
| TIL | IGLV4-69 | 0.695 | 1.53E-59 |
| TIL | IGLV1-47 | 0.695 | 1.55E-59 |
| TIL | IGHV1-24 | 0.695 | 1.57E-59 |
| TIL | PSMB8-AS1 | 0.694 | 2.06E-59 |
| TIL | AC007384.1 | 0.693 | 4.48E-59 |
| TIL | IGKV1-16 | 0.692 | 6.41E-59 |
| TIL | IGHV3-69-1 | 0.692 | 6.96E-59 |
| TIL | AC004585.1 | 0.692 | 7.49E-59 |
| TIL | WARS1 | 0.692 | 8.65E-59 |
| TIL | CD22 | 0.691 | 1.30E-58 |
| TIL | PIK3CD-AS1 | 0.691 | 1.42E-58 |
| TIL | S100B | 0.690 | 1.68E-58 |
| TIL | IGHV4-61 | 0.690 | 1.90E-58 |
| TIL | LINC02528 | 0.690 | 2.04E-58 |
| TIL | GPR55 | 0.690 | 2.16E-58 |
| TIL | MS4A7 | 0.689 | 2.82E-58 |
| TIL | LINC00892 | 0.689 | 2.91E-58 |
| TIL | HRH2 | 0.689 | 3.14E-58 |
| TIL | TMEM156 | 0.689 | 3.15E-58 |
| TIL | P2RY12 | 0.689 | 3.23E-58 |
| TIL | LINC01480 | 0.689 | 3.62E-58 |
| TIL | HSD11B1 | 0.689 | 3.73E-58 |
| TIL | LINC01684 | 0.689 | 4.03E-58 |
| TIL | HLA-DMA | 0.689 | 0.00E+00 |
| TIL | NUGGC | 0.688 | 4.81E-58 |
| TIL | LPXN | 0.688 | 5.28E-58 |
| TIL | IGKV1-9 | 0.688 | 7.48E-58 |
| TIL | NEXN | 0.688 | 7.70E-58 |
| TIL | TRBV14 | 0.687 | 8.29E-58 |
| TIL | GIMAP8 | 0.687 | 8.32E-58 |
| TIL | ICAM1 | 0.687 | 0.00E+00 |
| TIL | SPIB | 0.687 | 1.05E-57 |
| TIL | C2 | 0.687 | 1.15E-57 |
| TIL | GIMAP6 | 0.687 | 1.26E-57 |
| TIL | FPR2 | 0.687 | 1.26E-57 |
| TIL | VAV1 | 0.686 | 1.79E-57 |
| TIL | DTHD1 | 0.686 | 1.86E-57 |
| TIL | LILRA4 | 0.686 | 1.89E-57 |
| TIL | IGHV1-2 | 0.686 | 2.20E-57 |
| TIL | ARRDC5 | 0.685 | 2.41E-57 |
| TIL | IGLV3-9 | 0.685 | 3.08E-57 |
| TIL | EML4-AS1 | 0.685 | 3.36E-57 |
| TIL | IGHV4-4 | 0.685 | 3.76E-57 |
| TIL | TRAV5 | 0.684 | 4.44E-57 |
| TIL | HS3ST3B1 | 0.684 | 5.30E-57 |
| TIL | ST8SIA4 | 0.684 | 5.69E-57 |
| TIL | AC243829.4 | 0.683 | 7.22E-57 |
| TIL | AL354833.2 | 0.683 | 8.96E-57 |
| TIL | AL133467.1 | 0.683 | 9.27E-57 |
| TIL | IGKV1-17 | 0.682 | 1.18E-56 |
| TIL | GPR82 | 0.682 | 1.18E-56 |
| TIL | TRAV1-2 | 0.682 | 1.21E-56 |
| TIL | IGHA1 | 0.682 | 0.00E+00 |
| TIL | AL139125.1 | 0.682 | 1.78E-56 |
| TIL | IGHV1-69 | 0.681 | 2.27E-56 |
| TIL | IGHV4-28 | 0.681 | 2.40E-56 |
| TIL | SERPINA1 | 0.681 | 0.00E+00 |
| TIL | SIGLEC8 | 0.681 | 2.59E-56 |
| TIL | IGKV1-27 | 0.681 | 2.74E-56 |
| TIL | C5AR2 | 0.681 | 2.95E-56 |
| TIL | AC022706.1 | 0.680 | 3.54E-56 |
| TIL | MEFV | 0.679 | 5.53E-56 |
| TIL | CCDC141 | 0.678 | 8.40E-56 |
| TIL | IL18BP | 0.678 | 0.00E+00 |
| TIL | ANKRD44 | 0.678 | 1.08E-55 |
| TIL | ISG20 | 0.677 | 1.50E-55 |
| TIL | CLNK | 0.677 | 1.56E-55 |
| TIL | LINC02099 | 0.677 | 1.73E-55 |
| TIL | PLCB2 | 0.677 | 1.83E-55 |
| TIL | TRAV39 | 0.676 | 2.29E-55 |
| TIL | SEPTIN1 | 0.676 | 2.66E-55 |
| TIL | VENTX | 0.676 | 2.71E-55 |
| TIL | C4B | 0.676 | 2.94E-55 |
| TIL | CARD16 | 0.675 | 3.81E-55 |
| TIL | CCL13 | 0.675 | 4.97E-55 |
| TIL | PLD4 | 0.674 | 6.47E-55 |
| TIL | VIM | 0.674 | 0.00E+00 |
| TIL | TGM2 | 0.674 | 0.00E+00 |
| TIL | HLA-E | 0.673 | 0.00E+00 |
| TIL | EPSTI1 | 0.673 | 1.65E-54 |
| TIL | TM6SF1 | 0.672 | 1.78E-54 |
| TIL | TCL1A | 0.672 | 1.94E-54 |
| TIL | TRIM22 | 0.672 | 2.15E-54 |
| TIL | BIRC3 | 0.671 | 0.00E+00 |
| TIL | CD70 | 0.671 | 3.07E-54 |
| TIL | IGKV1D-39 | 0.671 | 4.14E-54 |
| TIL | IGKV1D-8 | 0.671 | 4.37E-54 |
| TIL | CEBPE | 0.670 | 5.36E-54 |
| TIL | SOCS1 | 0.669 | 0.00E+00 |
| TIL | IGHV3-66 | 0.669 | 9.87E-54 |
| TIL | HLA-DQB1-AS1 | 0.669 | 1.15E-53 |
| TIL | IDO2 | 0.669 | 1.15E-53 |
| TIL | ANTXRLP1 | 0.669 | 1.16E-53 |
| TIL | IGLV2-8 | 0.668 | 1.38E-53 |
| TIL | IGHV4-55 | 0.668 | 1.39E-53 |
| TIL | PARP15 | 0.668 | 1.48E-53 |
| TIL | CD1D | 0.667 | 2.00E-53 |
| TIL | TRGV4 | 0.667 | 2.04E-53 |
| TIL | IGKV3D-20 | 0.667 | 2.30E-53 |
| TIL | VNN1 | 0.667 | 2.62E-53 |
| TIL | HLA-F | 0.667 | 0.00E+00 |
| TIL | SLC24A4 | 0.667 | 2.98E-53 |
| TIL | TYMP | 0.666 | 0.00E+00 |
| TIL | TMEM106A | 0.666 | 3.49E-53 |
| TIL | IGHG4 | 0.666 | 0.00E+00 |
| TIL | CCL2 | 0.666 | 0.00E+00 |
| TIL | NCR1 | 0.666 | 4.35E-53 |
| TIL | CD300E | 0.666 | 4.66E-53 |
| TIL | FCN1 | 0.665 | 5.59E-53 |
| TIL | NTRK1 | 0.664 | 9.25E-53 |
| TIL | AC138207.5 | 0.664 | 0.00E+00 |
| TIL | NRROS | 0.664 | 1.05E-52 |
| TIL | PLA2G4C | 0.664 | 1.28E-52 |
| TIL | HLA-DQB2 | 0.663 | 0.00E+00 |
| TIL | SUSD3 | 0.663 | 1.56E-52 |
| TIL | LINC01150 | 0.663 | 2.15E-52 |
| TIL | IL12B | 0.662 | 3.06E-52 |
| TIL | IGLV7-43 | 0.662 | 3.31E-52 |
| TIL | IGHJ4 | 0.661 | 3.47E-52 |
| TIL | AC110611.2 | 0.661 | 3.65E-52 |
| TIL | IGKJ5 | 0.661 | 3.71E-52 |
| TIL | RTN1 | 0.661 | 3.92E-52 |
| TIL | AC005224.3 | 0.661 | 3.98E-52 |
| TIL | P2RY14 | 0.661 | 4.87E-52 |
| TIL | HLA-B | 0.660 | 0.00E+00 |
| TIL | TRAV8-1 | 0.660 | 6.68E-52 |
| TIL | IGHV2-70 | 0.660 | 8.00E-52 |
| TIL | FCRL1 | 0.660 | 8.08E-52 |
| TIL | IL15RA | 0.659 | 0.00E+00 |
| TIL | TRBJ1-5 | 0.659 | 1.13E-51 |
| TIL | IGKV3D-11 | 0.659 | 1.37E-51 |
| TIL | TLR7 | 0.658 | 1.46E-51 |
| TIL | LINC02084 | 0.658 | 1.53E-51 |
| TIL | MMP25 | 0.658 | 1.95E-51 |
| TIL | CYSLTR1 | 0.657 | 3.12E-51 |
| TIL | AC096667.1 | 0.656 | 3.76E-51 |
| TIL | IGHJ3 | 0.656 | 3.78E-51 |
| TIL | CYSLTR2 | 0.656 | 4.34E-51 |
| TIL | GLIPR1 | 0.656 | 4.44E-51 |
| TIL | IGKV1OR2-108 | 0.656 | 4.78E-51 |
| TIL | APOE | 0.656 | 0.00E+00 |
| TIL | AXL | 0.655 | 6.69E-51 |
| TIL | GLT1D1 | 0.655 | 7.12E-51 |
| TIL | C1orf54 | 0.654 | 9.27E-51 |
| TIL | ACP5 | 0.654 | 0.00E+00 |
| TIL | TREML1 | 0.654 | 9.85E-51 |
| TIL | RSPO3 | 0.654 | 1.07E-50 |
| TIL | IRF1 | 0.654 | 0.00E+00 |
| TIL | MIR223HG | 0.654 | 1.38E-50 |
| TIL | RASGRP2 | 0.654 | 1.40E-50 |
| TIL | IGLV5-45 | 0.653 | 1.56E-50 |
| TIL | ASB2 | 0.653 | 1.62E-50 |
| TIL | P2RX1 | 0.653 | 1.68E-50 |
| TIL | AC021188.1 | 0.653 | 1.72E-50 |
| TIL | IGKV3D-15 | 0.653 | 1.78E-50 |
| TIL | C19orf38 | 0.652 | 2.60E-50 |
| TIL | NAIPP1 | 0.652 | 2.78E-50 |
| TIL | AC010247.2 | 0.652 | 2.81E-50 |
| TIL | RAB37 | 0.652 | 3.02E-50 |
| TIL | GBP4 | 0.651 | 4.51E-50 |
| TIL | F13A1 | 0.651 | 5.05E-50 |
| TIL | L3MBTL4-AS1 | 0.651 | 5.17E-50 |
| TIL | SECTM1 | 0.651 | 0.00E+00 |
| TIL | BATF2 | 0.651 | 5.24E-50 |
| TIL | IGKV3OR2-268 | 0.651 | 5.62E-50 |
| TIL | NCF4 | 0.650 | 8.53E-50 |
| TIL | DNAJC5B | 0.650 | 8.75E-50 |
| TIL | IGHV3-62 | 0.649 | 9.46E-50 |
| TIL | TRAV13-2 | 0.649 | 9.99E-50 |
| TIL | LINC01914 | 0.649 | 1.04E-49 |
| TIL | IGHV3-73 | 0.649 | 1.21E-49 |
| TIL | GFI1 | 0.649 | 1.23E-49 |
| TIL | SH2B3 | 0.648 | 1.56E-49 |
| TIL | TRDV1 | 0.648 | 1.58E-49 |
| TIL | SP110 | 0.648 | 1.65E-49 |
| TIL | MATK | 0.648 | 1.70E-49 |
| TIL | C4A | 0.648 | 1.84E-49 |
| TIL | CLEC4A | 0.648 | 2.18E-49 |
| TIL | AL162414.1 | 0.647 | 2.82E-49 |
| TIL | C3 | 0.647 | 0.00E+00 |
| TIL | ANKRD36BP2 | 0.647 | 3.24E-49 |
| TIL | PIP4K2A | 0.647 | 3.48E-49 |
| TIL | IGKV2-24 | 0.646 | 3.63E-49 |
| TIL | AC015911.7 | 0.646 | 3.74E-49 |
| TIL | TBC1D27P | 0.646 | 4.04E-49 |
| TIL | LINC01146 | 0.646 | 4.32E-49 |
| TIL | AC011899.2 | 0.646 | 4.35E-49 |
| TIL | LINC00528 | 0.646 | 4.94E-49 |
| TIL | IGKV2D-29 | 0.646 | 5.31E-49 |
| TIL | LINC02285 | 0.646 | 5.39E-49 |
| TIL | C16orf54 | 0.645 | 6.25E-49 |
| TIL | TRAV25 | 0.645 | 6.82E-49 |
| TIL | AF127936.1 | 0.644 | 9.04E-49 |
| TIL | TRGV2 | 0.644 | 9.67E-49 |
| TIL | LINC02273 | 0.644 | 1.00E-48 |
| TIL | IGHJ5 | 0.644 | 1.06E-48 |
| TIL | TRBV24-1 | 0.644 | 1.24E-48 |
| TIL | XIRP1 | 0.643 | 1.42E-48 |
| TIL | PNOC | 0.643 | 1.47E-48 |
| TIL | RFTN1 | 0.643 | 0.00E+00 |
| TIL | KIR2DL4 | 0.643 | 1.59E-48 |
| TIL | AC012236.1 | 0.643 | 1.63E-48 |
| TIL | FCGR2C | 0.643 | 1.76E-48 |
| TIL | IGKJ3 | 0.643 | 1.84E-48 |
| TIL | MILR1 | 0.642 | 2.67E-48 |
| TIL | TRAV10 | 0.642 | 2.83E-48 |
| TIL | IGLV1-36 | 0.642 | 2.94E-48 |
| TIL | IGLV3-16 | 0.642 | 3.14E-48 |
| TIL | IGKV5-2 | 0.641 | 3.57E-48 |
| TIL | PTPRN2-AS1 | 0.641 | 3.66E-48 |
| TIL | IGLV2-5 | 0.641 | 3.76E-48 |
| TIL | IGKV1D-33 | 0.641 | 3.82E-48 |
| TIL | IFITM3 | 0.641 | 0.00E+00 |
| TIL | ENTPD1 | 0.641 | 4.84E-48 |
| TIL | NNMT | 0.640 | 0.00E+00 |
| TIL | IGHV3-13 | 0.640 | 5.71E-48 |
| TIL | TRBV12-3 | 0.640 | 5.98E-48 |
| TIL | LINC02785 | 0.640 | 6.29E-48 |
| TIL | IGLV8-61 | 0.640 | 7.01E-48 |
| TIL | IGLV3-27 | 0.640 | 7.20E-48 |
| TIL | TRBJ1-6 | 0.639 | 9.07E-48 |
| TIL | TRGV3 | 0.639 | 9.38E-48 |
| TIL | IGLV2-18 | 0.639 | 9.50E-48 |
| TIL | STAT1 | 0.639 | 0.00E+00 |
| TIL | IGHV3-71 | 0.639 | 9.60E-48 |
| TIL | HLA-DQA2 | 0.639 | 9.74E-48 |
| TIL | DBH-AS1 | 0.639 | 1.02E-47 |
| TIL | AC079209.1 | 0.639 | 1.12E-47 |
| TIL | TRGV9 | 0.638 | 1.58E-47 |
| TIL | TRAV29DV5 | 0.638 | 1.59E-47 |
| TIL | CPVL | 0.638 | 1.82E-47 |
| TIL | AC022126.1 | 0.637 | 2.10E-47 |
| TIL | TSPAN4 | 0.637 | 2.15E-47 |
| TIL | IGLV9-49 | 0.637 | 2.16E-47 |
| TIL | ADGRE4P | 0.637 | 2.27E-47 |
| TIL | IGHV3-43 | 0.637 | 2.76E-47 |
| TIL | DLEU7 | 0.636 | 4.31E-47 |
| TIL | KIR3DL2 | 0.636 | 4.35E-47 |
| TIL | IGHV1OR15-2 | 0.635 | 4.43E-47 |
| TIL | GPBAR1 | 0.635 | 4.51E-47 |
| TIL | PTPRJ | 0.635 | 4.78E-47 |
| TIL | CLEC17A | 0.634 | 6.71E-47 |
| TIL | IGHV2-26 | 0.634 | 6.73E-47 |
| TIL | MSC | 0.634 | 0.00E+00 |
| TIL | TREML2 | 0.634 | 8.63E-47 |
| TIL | TRAF1 | 0.633 | 1.06E-46 |
| TIL | MMP9 | 0.633 | 1.10E-46 |
| TIL | SFMBT2 | 0.633 | 1.11E-46 |
| TIL | AC124014.1 | 0.633 | 1.22E-46 |
| TIL | IGLV3-10 | 0.633 | 1.25E-46 |
| TIL | TLR6 | 0.633 | 1.57E-46 |
| TIL | POU2F2 | 0.632 | 2.00E-46 |
| TIL | IL27 | 0.632 | 2.01E-46 |
| TIL | TRBV5-5 | 0.632 | 2.09E-46 |
| TIL | TRAV38-1 | 0.632 | 2.17E-46 |
| TIL | LCT-AS1 | 0.631 | 3.05E-46 |
| TIL | IGKJ4 | 0.631 | 3.10E-46 |
| TIL | IFITM1 | 0.630 | 0.00E+00 |
| TIL | TRBJ1-4 | 0.630 | 4.41E-46 |
| TIL | IGKV1-8 | 0.630 | 4.70E-46 |
| TIL | TRAV26-2 | 0.630 | 4.77E-46 |
| TIL | USP30-AS1 | 0.630 | 5.12E-46 |
| TIL | ADGRE1 | 0.630 | 5.38E-46 |
| TIL | IGHV3-20 | 0.630 | 5.46E-46 |
| TIL | OSM | 0.629 | 5.82E-46 |
| TIL | TRAV6 | 0.629 | 6.70E-46 |
| TIL | TNFSF4 | 0.629 | 7.03E-46 |
| TIL | IGHV3-74 | 0.628 | 9.25E-46 |
| TIL | ADAM19 | 0.628 | 1.13E-45 |
| TIL | IGKV3-7 | 0.628 | 1.25E-45 |
| TIL | KCNAB2 | 0.627 | 1.40E-45 |
| TIL | KIAA1755 | 0.627 | 1.89E-45 |
| TIL | IGHV1-58 | 0.626 | 1.99E-45 |
| TIL | CHI3L1 | 0.626 | 0.00E+00 |
| TIL | CMAHP | 0.626 | 2.02E-45 |
| TIL | APBB1IP | 0.626 | 0.00E+00 |
| TIL | TAP1 | 0.626 | 0.00E+00 |
| TIL | JAZF1 | 0.626 | 2.67E-45 |
| TIL | IGKV1-33 | 0.625 | 3.15E-45 |
| TIL | TRAV24 | 0.625 | 4.48E-45 |
| TIL | POU2AF1 | 0.624 | 5.22E-45 |
| TIL | GPSM3 | 0.624 | 6.47E-45 |
| TIL | OLFML3 | 0.623 | 7.64E-45 |
| TIL | IGHD | 0.623 | 7.98E-45 |
| TIL | AC135068.3 | 0.623 | 8.59E-45 |
| TIL | IGKV6-21 | 0.623 | 9.22E-45 |
| TIL | AC099524.1 | 0.623 | 9.72E-45 |
| TIL | AL121985.1 | 0.622 | 1.08E-44 |
| TIL | SYT11 | 0.622 | 1.12E-44 |
| TIL | CNR2 | 0.622 | 1.18E-44 |
| TIL | LINC00968 | 0.622 | 1.20E-44 |
| TIL | AC007877.1 | 0.622 | 1.49E-44 |
| TIL | CD19 | 0.621 | 1.64E-44 |
| TIL | RAB27A | 0.621 | 0.00E+00 |
| TIL | ZNF80 | 0.621 | 1.91E-44 |
| TIL | SUCNR1 | 0.621 | 2.11E-44 |
| TIL | GPR25 | 0.621 | 2.16E-44 |
| TIL | TRBV6-6 | 0.620 | 2.42E-44 |
| TIL | SLC2A5 | 0.620 | 2.57E-44 |
| TIL | ZBED2 | 0.620 | 2.97E-44 |
| TIL | NTNG2 | 0.620 | 3.16E-44 |
| TIL | KCTD12 | 0.620 | 0.00E+00 |
| TIL | UBE2L6 | 0.619 | 0.00E+00 |
| TIL | TRBV10-2 | 0.619 | 3.65E-44 |
| TIL | DCSTAMP | 0.619 | 3.67E-44 |
| TIL | ZMYND15 | 0.619 | 3.84E-44 |
| TIL | AC244502.1 | 0.619 | 3.86E-44 |
| TIL | CD274 | 0.619 | 3.98E-44 |
| TIL | LINC01215 | 0.619 | 3.99E-44 |
| TIL | IGLJ2 | 0.619 | 4.26E-44 |
| TIL | GGT5 | 0.619 | 0.00E+00 |
| TIL | KIF19 | 0.619 | 4.72E-44 |
| TIL | TFAP2E-AS1 | 0.619 | 4.88E-44 |
| TIL | AC015819.1 | 0.618 | 5.55E-44 |
| TIL | LINC02362 | 0.618 | 6.36E-44 |
| TIL | P2RX7 | 0.618 | 7.39E-44 |
| TIL | IGHV1-3 | 0.618 | 7.63E-44 |
| TIL | MCOLN2 | 0.617 | 8.76E-44 |
| TIL | SIRPA | 0.617 | 0.00E+00 |
| TIL | CLEC7A | 0.617 | 1.10E-43 |
| TIL | C9orf139 | 0.617 | 1.14E-43 |
| TIL | IGLV7-46 | 0.616 | 1.60E-43 |
| TIL | IGHJ3P | 0.615 | 1.81E-43 |
| TIL | MACORIS | 0.615 | 2.10E-43 |
| TIL | GPR68 | 0.615 | 0.00E+00 |
| TIL | TRBV25-1 | 0.615 | 2.31E-43 |
| TIL | AL356750.1 | 0.615 | 2.47E-43 |
| TIL | GRAMD1B | 0.614 | 2.61E-43 |
| TIL | IGKV1D-43 | 0.614 | 2.85E-43 |
| TIL | IGKV2D-40 | 0.614 | 3.07E-43 |
| TIL | XXYLT1-AS2 | 0.614 | 3.12E-43 |
| TIL | PTGS1 | 0.614 | 3.15E-43 |
| TIL | IGHV3OR16-9 | 0.614 | 3.30E-43 |
| TIL | SIGLEC11 | 0.614 | 3.80E-43 |
| TIL | CCL21 | 0.613 | 3.91E-43 |
| TIL | TMEM140 | 0.613 | 4.07E-43 |
| TIL | TNFAIP6 | 0.613 | 4.78E-43 |
| TIL | IGHV1OR15-9 | 0.613 | 4.99E-43 |
| TIL | IL15 | 0.612 | 6.26E-43 |
| TIL | LRRK2 | 0.612 | 6.84E-43 |
| TIL | SAMD9L | 0.612 | 0.00E+00 |
| TIL | IFNG-AS1 | 0.612 | 7.76E-43 |
| TIL | AC034199.1 | 0.612 | 7.90E-43 |
| TIL | CLIC2 | 0.612 | 0.00E+00 |
| TIL | AC142381.1 | 0.611 | 1.00E-42 |
| TIL | TRBC2 | 0.611 | 0.00E+00 |
| TIL | ARHGAP45 | 0.611 | 0.00E+00 |
| TIL | IGKV1D-16 | 0.611 | 1.17E-42 |
| TIL | TRBV6-1 | 0.611 | 1.20E-42 |
| TIL | SARDH | 0.611 | 1.25E-42 |
| TIL | SYTL3 | 0.610 | 1.27E-42 |
| TIL | CPNE5 | 0.610 | 1.45E-42 |
| TIL | AC008105.3 | 0.610 | 1.59E-42 |
| TIL | RAC2 | 0.610 | 0.00E+00 |
| TIL | MIR4539 | 0.609 | 2.20E-42 |
| TIL | TRAV19 | 0.609 | 2.50E-42 |
| TIL | IGLC7 | 0.609 | 2.56E-42 |
| TIL | BIN1 | 0.609 | 0.00E+00 |
| TIL | SEPTIN6 | 0.608 | 2.83E-42 |
| TIL | PTGER2 | 0.608 | 2.84E-42 |
| TIL | IGKV1OR2-6 | 0.607 | 4.28E-42 |
| TIL | ANKRD55 | 0.607 | 4.29E-42 |
| TIL | PDE6G | 0.607 | 4.88E-42 |
| TIL | CCDC80 | 0.607 | 5.48E-42 |
| TIL | CAVIN1 | 0.607 | 0.00E+00 |
| TIL | TOX | 0.607 | 5.73E-42 |
| TIL | FAM20C | 0.607 | 0.00E+00 |
| TIL | ARID5A | 0.607 | 0.00E+00 |
| TIL | SDS | 0.605 | 8.96E-42 |
| TIL | IGHV3OR16-13 | 0.605 | 9.76E-42 |
| TIL | IGHV3-63 | 0.605 | 1.17E-41 |
| TIL | CRYBB1 | 0.605 | 1.20E-41 |
| TIL | TRBV21-1 | 0.605 | 1.22E-41 |
| TIL | CD244 | 0.604 | 1.47E-41 |
| TIL | IL6 | 0.604 | 1.50E-41 |
| TIL | GFPT2 | 0.604 | 1.60E-41 |
| TIL | DOCK11 | 0.604 | 1.64E-41 |
| TIL | IGHV3-64D | 0.604 | 1.89E-41 |
| TIL | BTN3A3 | 0.603 | 0.00E+00 |
| TIL | LINC02391 | 0.602 | 3.01E-41 |
| TIL | LINC02694 | 0.602 | 3.42E-41 |
| TIL | CIBAR2 | 0.602 | 3.52E-41 |
| TIL | CLEC6A | 0.602 | 3.75E-41 |
| TIL | NME8 | 0.602 | 3.80E-41 |
| TIL | STARD8 | 0.602 | 3.87E-41 |
| TIL | AC243962.1 | 0.602 | 4.08E-41 |
| TIL | IGLC6 | 0.601 | 4.78E-41 |
| TIL | PLAAT4 | 0.601 | 0.00E+00 |
| TIL | IGKV2-28 | 0.601 | 5.68E-41 |
| TIL | CCDC170 | 0.600 | 8.27E-41 |
| TIL | PATL2 | 0.600 | 8.46E-41 |
| TIL | IGLV2-28 | 0.599 | 8.86E-41 |
| TIL | VMO1 | 0.599 | 0.00E+00 |
| TIL | AC136428.1 | 0.599 | 9.05E-41 |
| TIL | CEACAM3 | 0.599 | 9.56E-41 |
| TIL | CPA3 | 0.599 | 9.66E-41 |
| TIL | RPL32P1 | 0.599 | 9.81E-41 |
| TIL | LINC02539 | 0.599 | 1.19E-40 |
| TIL | CLEC5A | 0.599 | 1.23E-40 |
| TIL | CTSK | 0.599 | 1.26E-40 |
| TIL | GAS7 | 0.598 | 1.59E-40 |
| TIL | ASGR2 | 0.598 | 1.60E-40 |
| TIL | PRRX1 | 0.598 | 1.73E-40 |
| TIL | IGLV5-48 | 0.597 | 1.91E-40 |
| TIL | IFITM2 | 0.597 | 0.00E+00 |
| TIL | LYL1 | 0.597 | 0.00E+00 |
| TIL | IGHV1-67 | 0.596 | 3.27E-40 |
| TIL | COLEC12 | 0.596 | 3.57E-40 |
| TIL | TAP2 | 0.596 | 0.00E+00 |
| TIL | CTSB | 0.595 | 0.00E+00 |
| TIL | CPXM1 | 0.595 | 4.89E-40 |
| TIL | AC134879.2 | 0.595 | 5.03E-40 |
| TIL | GNB4 | 0.595 | 5.08E-40 |
| TIL | SMAP2 | 0.594 | 0.00E+00 |
| TIL | TRAV35 | 0.594 | 7.28E-40 |
| TIL | HLA-A | 0.594 | 0.00E+00 |
| TIL | HTRA4 | 0.593 | 9.76E-40 |
| TIL | TPSAB1 | 0.593 | 9.98E-40 |
| TIL | IGHV3OR16-8 | 0.593 | 9.99E-40 |
| TIL | IGHV3-72 | 0.592 | 1.21E-39 |
| TIL | IFIT3 | 0.592 | 0.00E+00 |
| TIL | SOCAR | 0.591 | 1.75E-39 |
| TIL | FCER2 | 0.591 | 1.83E-39 |
| TIL | IGKV1D-12 | 0.591 | 1.93E-39 |
| TIL | KCNA2 | 0.591 | 2.01E-39 |
| TIL | RASGRP1 | 0.591 | 2.06E-39 |
| TIL | GLT8D2 | 0.591 | 0.00E+00 |
| TIL | RIN3 | 0.591 | 2.09E-39 |
| TIL | HEG1 | 0.591 | 0.00E+00 |
| TIL | TRGV8 | 0.590 | 2.80E-39 |
| TIL | COL6A2 | 0.590 | 0.00E+00 |
| TIL | LINC00877 | 0.590 | 3.48E-39 |
| TIL | IGSF21 | 0.590 | 3.49E-39 |
| TIL | CCL3L3 | 0.589 | 3.69E-39 |
| TIL | AC027559.1 | 0.589 | 4.26E-39 |
| TIL | CHI3L2 | 0.589 | 4.39E-39 |
| TIL | MARCO | 0.589 | 4.65E-39 |
| TIL | ACOD1 | 0.589 | 4.72E-39 |
| TIL | ARHGAP31 | 0.588 | 5.83E-39 |
| TIL | ELMO1 | 0.588 | 5.84E-39 |
| TIL | ERFL | 0.588 | 6.21E-39 |
| TIL | ANKRD29 | 0.587 | 9.29E-39 |
| TIL | AC084871.3 | 0.587 | 9.46E-39 |
| TIL | LINC00402 | 0.586 | 1.28E-38 |
| TIL | GTSF1L | 0.586 | 1.52E-38 |
| TIL | SUGCT-AS1 | 0.586 | 1.53E-38 |
| TIL | RIPOR2 | 0.585 | 1.61E-38 |
| TIL | CCL7 | 0.585 | 1.73E-38 |
| TIL | KMO | 0.585 | 1.76E-38 |
| TIL | FAS | 0.585 | 1.81E-38 |
| TIL | MARCHF1 | 0.585 | 2.02E-38 |
| TIL | FMOD | 0.584 | 0.00E+00 |
| TIL | FMNL3 | 0.584 | 2.93E-38 |
| TIL | LINC01781 | 0.584 | 3.05E-38 |
| TIL | IGLV3-12 | 0.583 | 3.16E-38 |
| TIL | AC110995.1 | 0.583 | 3.27E-38 |
| TIL | TLDC2 | 0.583 | 3.48E-38 |
| TIL | MSC-AS1 | 0.583 | 3.49E-38 |
| TIL | CD1B | 0.583 | 3.78E-38 |
| TIL | ATP10A | 0.583 | 4.07E-38 |
| TIL | ADAMTS2 | 0.583 | 4.08E-38 |
| TIL | LGALS17A | 0.582 | 4.52E-38 |
| TIL | PECAM1 | 0.582 | 0.00E+00 |
| TIL | TRBJ1-1 | 0.582 | 5.25E-38 |
| TIL | AMPD1 | 0.582 | 5.48E-38 |
| TIL | FCRL4 | 0.581 | 6.90E-38 |
| TIL | KLRC2 | 0.581 | 8.09E-38 |
| TIL | FAIM2 | 0.581 | 8.58E-38 |
| TIL | IGLV2-34 | 0.581 | 9.12E-38 |
| TIL | CLIC5 | 0.580 | 9.64E-38 |
| TIL | SIGLEC12 | 0.580 | 1.00E-37 |
| TIL | FCMR | 0.580 | 0.00E+00 |
| TIL | MT2A | 0.580 | 0.00E+00 |
| TIL | CCDC71L | 0.580 | 1.19E-37 |
| TIL | SCN1B | 0.579 | 1.46E-37 |
| TIL | CALHM5 | 0.579 | 1.50E-37 |
| TIL | MSN | 0.579 | 0.00E+00 |
| TIL | AC034238.1 | 0.579 | 1.60E-37 |
| TIL | IGHJ6 | 0.578 | 1.89E-37 |
| TIL | ABI3BP | 0.578 | 1.91E-37 |
| TIL | PTGIR | 0.578 | 2.08E-37 |
| TIL | CCN4 | 0.578 | 2.11E-37 |
| TIL | HVCN1 | 0.578 | 2.14E-37 |
| TIL | IGHJ2 | 0.578 | 2.27E-37 |
| TIL | LINC02576 | 0.578 | 2.35E-37 |
| TIL | TWIST2 | 0.577 | 2.82E-37 |
| TIL | SIRPB1 | 0.577 | 2.86E-37 |
| TIL | IL22RA2 | 0.577 | 3.09E-37 |
| TIL | PTAFR | 0.577 | 3.19E-37 |
| TIL | LINC00582 | 0.577 | 3.26E-37 |
| TIL | MMP2-AS1 | 0.577 | 3.40E-37 |
| TIL | RASA3 | 0.577 | 0.00E+00 |
| TIL | RAB3IL1 | 0.576 | 3.94E-37 |
| TIL | CADM3 | 0.576 | 4.11E-37 |
| TIL | PALM2AKAP2 | 0.576 | 4.21E-37 |
| TIL | IFIT2 | 0.576 | 4.23E-37 |
| TIL | IGHV2-70D | 0.575 | 5.47E-37 |
| TIL | FAP | 0.575 | 6.01E-37 |
| TIL | CCN1 | 0.575 | 0.00E+00 |
| TIL | ARHGAP30 | 0.575 | 6.61E-37 |
| TIL | PPP1R18 | 0.575 | 0.00E+00 |
| TIL | TSHR | 0.574 | 8.41E-37 |
| TIL | SYNE3 | 0.574 | 8.47E-37 |
| TIL | KCNMB1 | 0.574 | 8.90E-37 |
| TIL | RAB31 | 0.574 | 0.00E+00 |
| TIL | EMILIN1 | 0.574 | 0.00E+00 |
| TIL | TMEM71 | 0.574 | 9.36E-37 |
| TIL | OLFML1 | 0.574 | 1.02E-36 |
| TIL | AC064805.1 | 0.573 | 1.21E-36 |
| TIL | PLEKHA3P1 | 0.573 | 1.28E-36 |
| TIL | HIC1 | 0.573 | 0.00E+00 |
| TIL | AC246787.2 | 0.573 | 1.38E-36 |
| TIL | AC079793.1 | 0.573 | 1.43E-36 |
| TIL | AL357054.4 | 0.573 | 1.48E-36 |
| TIL | PALD1 | 0.572 | 1.71E-36 |
| TIL | AC108134.3 | 0.572 | 1.72E-36 |
| TIL | IGHV3-60 | 0.572 | 1.74E-36 |
| TIL | IGHV3OR15-7 | 0.572 | 1.77E-36 |
| TIL | CXCL12 | 0.572 | 0.00E+00 |
| TIL | AEBP1 | 0.572 | 0.00E+00 |
| TIL | DCN | 0.572 | 0.00E+00 |
| TIL | FCAR | 0.572 | 1.87E-36 |
| TIL | DDR2 | 0.572 | 0.00E+00 |
| TIL | AC136475.2 | 0.571 | 2.10E-36 |
| TIL | GPR31 | 0.571 | 2.39E-36 |
| TIL | IGHV3-35 | 0.571 | 2.62E-36 |
| TIL | STK17B | 0.571 | 2.88E-36 |
| TIL | GRAP | 0.570 | 3.17E-36 |
| TIL | CD200R1 | 0.570 | 3.30E-36 |
| TIL | DNASE1L3 | 0.570 | 3.69E-36 |
| TIL | MEF2C | 0.570 | 4.09E-36 |
| TIL | IRF1-AS1 | 0.569 | 4.33E-36 |
| TIL | TGFB3 | 0.569 | 4.44E-36 |
| TIL | COL15A1 | 0.569 | 0.00E+00 |
| TIL | PAPSS2 | 0.569 | 4.92E-36 |
| TIL | GPC6 | 0.569 | 5.22E-36 |
| TIL | AC010883.2 | 0.569 | 5.32E-36 |
| TIL | TOX2 | 0.569 | 5.34E-36 |
| TIL | AC005224.2 | 0.569 | 5.51E-36 |
| TIL | AQP9 | 0.568 | 5.99E-36 |
| TIL | AC243960.3 | 0.568 | 6.21E-36 |
| TIL | DERL3 | 0.568 | 6.36E-36 |
| TIL | TIMD4 | 0.568 | 7.17E-36 |
| TIL | EGR2 | 0.568 | 7.29E-36 |
| TIL | IGHV3-64 | 0.568 | 7.60E-36 |
| TIL | IGKV2OR22-4 | 0.568 | 8.05E-36 |
| TIL | SLC11A1 | 0.568 | 8.17E-36 |
| TIL | CLMP | 0.567 | 8.63E-36 |
| TIL | RGS2 | 0.567 | 0.00E+00 |
| TIL | AL138930.1 | 0.567 | 8.83E-36 |
| TIL | IGKV1-13 | 0.567 | 9.42E-36 |
| TIL | AC090825.1 | 0.567 | 9.55E-36 |
| TIL | DPT | 0.567 | 9.75E-36 |
| TIL | MS4A14 | 0.567 | 1.12E-35 |
| TIL | COL23A1 | 0.566 | 1.15E-35 |
| TIL | MYO7A | 0.566 | 1.18E-35 |
| TIL | LCP1 | 0.566 | 0.00E+00 |
| TIL | KCNMA1 | 0.566 | 1.20E-35 |
| TIL | CNRIP1 | 0.566 | 1.21E-35 |
| TIL | NRP2 | 0.566 | 1.37E-35 |
| TIL | IFI35 | 0.566 | 0.00E+00 |
| TIL | PRXL2C | 0.566 | 1.55E-35 |
| TIL | IGHV3-19 | 0.565 | 1.96E-35 |
| TIL | IGLV10-54 | 0.564 | 2.41E-35 |
| TIL | CCND2 | 0.564 | 0.00E+00 |
| TIL | RAMP1 | 0.564 | 2.95E-35 |
| TIL | ODF3B | 0.564 | 0.00E+00 |
| TIL | BEST1 | 0.564 | 3.03E-35 |
| TIL | SYNPO | 0.563 | 3.25E-35 |
| TIL | FBN1 | 0.563 | 3.29E-35 |
| TIL | AL121933.2 | 0.563 | 3.42E-35 |
| TIL | CACNA2D4 | 0.563 | 3.47E-35 |
| TIL | PLEKHO1 | 0.563 | 3.53E-35 |
| TIL | CCL22 | 0.563 | 3.95E-35 |
| TIL | IGHV3-41 | 0.563 | 4.16E-35 |
| TIL | CXCR5 | 0.563 | 4.21E-35 |
| TIL | IGHV1-12 | 0.562 | 4.66E-35 |
| TIL | SH2D3C | 0.562 | 4.77E-35 |
| TIL | IGHV1OR21-1 | 0.562 | 4.96E-35 |
| TIL | IGHV1-45 | 0.562 | 5.03E-35 |
| TIL | EFEMP1 | 0.562 | 0.00E+00 |
| TIL | SENCR | 0.562 | 5.43E-35 |
| TIL | HLA-C | 0.562 | 0.00E+00 |
| TIL | LINGO3 | 0.562 | 5.87E-35 |
| TIL | AC104971.3 | 0.561 | 6.37E-35 |
| TIL | P2RY6 | 0.561 | 6.58E-35 |
| TIL | AC092580.4 | 0.561 | 7.11E-35 |
| TIL | LSP1 | 0.561 | 7.39E-35 |
| TIL | COL6A3 | 0.561 | 0.00E+00 |
| TIL | STING1 | 0.561 | 7.61E-35 |
| TIL | CCL24 | 0.561 | 7.67E-35 |
| TIL | ADAMTS7P4 | 0.560 | 1.01E-34 |
| TIL | LINC02812 | 0.560 | 1.07E-34 |
| TIL | SERPINB9 | 0.560 | 1.09E-34 |
| TIL | TNFRSF18 | 0.560 | 0.00E+00 |
| TIL | SULF1 | 0.560 | 1.14E-34 |
| TIL | KLRC1 | 0.560 | 1.15E-34 |
| TIL | STK10 | 0.560 | 1.15E-34 |
| TIL | FKBP5 | 0.559 | 1.19E-34 |
| TIL | IGKV2D-30 | 0.559 | 1.27E-34 |
| TIL | FBLN5 | 0.559 | 0.00E+00 |
| TIL | ALPK2 | 0.559 | 1.40E-34 |
| TIL | AL031733.2 | 0.559 | 1.54E-34 |
| TIL | TRGV5P | 0.559 | 1.56E-34 |
| TIL | RHOG | 0.559 | 1.57E-34 |
| TIL | PTCSC1 | 0.559 | 1.59E-34 |
| TIL | BNC2 | 0.558 | 1.78E-34 |
| TIL | CYLD | 0.558 | 1.79E-34 |
| TIL | IGKV1D-13 | 0.558 | 1.93E-34 |
| TIL | PREX1 | 0.558 | 1.98E-34 |
| TIL | IGKV2D-28 | 0.557 | 2.28E-34 |
| TIL | TMEM244 | 0.557 | 2.35E-34 |
| TIL | GIMAP2 | 0.557 | 0.00E+00 |
| TIL | CCN2 | 0.557 | 0.00E+00 |
| TIL | IGKV1D-27 | 0.557 | 2.63E-34 |
| TIL | AC245884.12 | 0.557 | 3.13E-34 |
| TIL | CSF3R | 0.556 | 3.56E-34 |
| TIL | COPDA1 | 0.556 | 3.74E-34 |
| TIL | AC093583.1 | 0.556 | 3.98E-34 |
| TIL | FGF7 | 0.556 | 4.04E-34 |
| TIL | GMFG | 0.556 | 0.00E+00 |
| TIL | FLNA | 0.556 | 0.00E+00 |
| TIL | LINC01678 | 0.556 | 4.30E-34 |
| TIL | Z95114.3 | 0.555 | 4.45E-34 |
| TIL | CALD1 | 0.555 | 0.00E+00 |
| TIL | PDGFRB | 0.555 | 4.88E-34 |
| TIL | AP003774.2 | 0.555 | 5.18E-34 |
| TIL | C1QTNF1 | 0.555 | 0.00E+00 |
| TIL | DPYD | 0.555 | 0.00E+00 |
| TIL | AL135818.1 | 0.554 | 6.05E-34 |
| TIL | CYTOR | 0.554 | 6.20E-34 |
| TIL | KCNJ5 | 0.554 | 6.31E-34 |
| TIL | HCP5 | 0.554 | 6.56E-34 |
| TIL | AC008760.2 | 0.554 | 7.17E-34 |
| TIL | PDPN | 0.553 | 1.01E-33 |
| TIL | FGR | 0.553 | 1.10E-33 |
| TIL | LINC00092 | 0.552 | 1.18E-33 |
| TIL | ADAMTS4 | 0.552 | 0.00E+00 |
| TIL | CHST11 | 0.552 | 1.27E-33 |
| TIL | IGHJ1 | 0.552 | 1.27E-33 |
| TIL | CD163L1 | 0.552 | 1.30E-33 |
| TIL | IGF1 | 0.552 | 1.33E-33 |
| TIL | DPF3 | 0.552 | 1.50E-33 |
| TIL | KIR2DL3 | 0.552 | 1.53E-33 |
| TIL | HCLS1 | 0.551 | 0.00E+00 |
| TIL | GREM1 | 0.551 | 1.97E-33 |
| TIL | MIR100HG | 0.551 | 2.00E-33 |
| TIL | MT1L | 0.551 | 2.11E-33 |
| TIL | RGS10 | 0.550 | 0.00E+00 |
| TIL | ZYX | 0.550 | 0.00E+00 |
| TIL | AC136628.3 | 0.550 | 2.48E-33 |
| TIL | RAMP3 | 0.550 | 0.00E+00 |
| TIL | IGHV3-52 | 0.550 | 2.68E-33 |
| TIL | TRIM61 | 0.550 | 2.76E-33 |
| TIL | CRISPLD2 | 0.549 | 0.00E+00 |
| TIL | CTSZ | 0.549 | 0.00E+00 |
| TIL | ADGRG3 | 0.549 | 3.35E-33 |
| TIL | AC107959.1 | 0.549 | 3.38E-33 |
| TIL | AL137186.1 | 0.549 | 3.39E-33 |
| TIL | AC093010.1 | 0.549 | 3.39E-33 |
| TIL | LINC02345 | 0.549 | 3.57E-33 |
| TIL | E2F3P1 | 0.549 | 3.78E-33 |
| TIL | LINC00243 | 0.549 | 3.86E-33 |
| TIL | TIMP2 | 0.549 | 0.00E+00 |
| TIL | FDCSP | 0.549 | 3.88E-33 |
| TIL | ACVRL1 | 0.548 | 4.30E-33 |
| TIL | AC096734.1 | 0.548 | 4.88E-33 |
| TIL | KIR3DX1 | 0.548 | 5.07E-33 |
| TIL | TTC16 | 0.548 | 5.10E-33 |
| TIL | TCN2 | 0.548 | 0.00E+00 |
| TIL | IGKV1OR2-11 | 0.548 | 5.40E-33 |
| TIL | TRAV1-1 | 0.548 | 5.50E-33 |
| TIL | TRBV23-1 | 0.548 | 5.53E-33 |
| TIL | RAB33A | 0.547 | 6.47E-33 |
| TIL | LILRP2 | 0.547 | 6.57E-33 |
| TIL | IGKV1OR22-5 | 0.547 | 7.18E-33 |
| TIL | HLA-DPA3 | 0.547 | 7.33E-33 |
| TIL | LINC00544 | 0.546 | 8.27E-33 |
| TIL | AP002954.1 | 0.546 | 1.01E-32 |
| TIL | AKNA | 0.545 | 1.10E-32 |
| TIL | CYBRD1 | 0.545 | 0.00E+00 |
| TIL | FILIP1L | 0.545 | 1.25E-32 |
| TIL | CCR6 | 0.545 | 1.43E-32 |
| TIL | SERPINF1 | 0.545 | 1.43E-32 |
| TIL | THBS1 | 0.544 | 0.00E+00 |
| TIL | COL16A1 | 0.544 | 0.00E+00 |
| TIL | STOM | 0.544 | 0.00E+00 |
| TIL | CREB5 | 0.544 | 1.80E-32 |
| TIL | SFRP2 | 0.543 | 2.05E-32 |
| TIL | TRAV27 | 0.543 | 2.48E-32 |
| TIL | ACTB | 0.543 | 0.00E+00 |
| TIL | AC145098.1 | 0.542 | 2.76E-32 |
| TIL | SLC2A3 | 0.542 | 2.89E-32 |
| TIL | CTSL | 0.542 | 0.00E+00 |
| TIL | TRGV5 | 0.542 | 2.97E-32 |
| TIL | AC010247.1 | 0.542 | 3.07E-32 |
| TIL | TRGV7 | 0.542 | 3.21E-32 |
| TIL | IFIH1 | 0.542 | 3.48E-32 |
| TIL | TNFAIP8L3 | 0.542 | 3.54E-32 |
| TIL | TMEM200A | 0.541 | 3.86E-32 |
| TIL | DYSF | 0.541 | 4.02E-32 |
| TIL | TRBV7-4 | 0.541 | 4.05E-32 |
| TIL | SIGLEC17P | 0.541 | 4.36E-32 |
| TIL | AC022296.2 | 0.541 | 4.71E-32 |
| TIL | CCL1 | 0.540 | 5.27E-32 |
| TIL | LINC01506 | 0.540 | 5.42E-32 |
| TIL | ISLR | 0.540 | 0.00E+00 |
| TIL | POM121L9P | 0.540 | 5.75E-32 |
| TIL | COL6A1 | 0.540 | 0.00E+00 |
| TIL | IGHV3-6 | 0.540 | 6.44E-32 |
| TIL | MAFB | 0.540 | 6.70E-32 |
| TIL | AC008033.3 | 0.539 | 8.11E-32 |
| TIL | ARHGAP22 | 0.539 | 8.66E-32 |
| TIL | TOGARAM2 | 0.538 | 9.45E-32 |
| TIL | SPATC1 | 0.538 | 9.57E-32 |
| TIL | CTSS | 0.538 | 0.00E+00 |
| TIL | BTN3A1 | 0.538 | 9.99E-32 |
| TIL | HTRA3 | 0.538 | 0.00E+00 |
| TIL | TPSB2 | 0.538 | 1.12E-31 |
| TIL | IGKV2-26 | 0.538 | 1.21E-31 |
| TIL | AC109446.2 | 0.538 | 1.25E-31 |
| TIL | SPHK1 | 0.538 | 1.25E-31 |
| TIL | IGKV2-29 | 0.537 | 1.31E-31 |
| TIL | IL6ST | 0.537 | 1.37E-31 |
| TIL | ZNF469 | 0.537 | 1.47E-31 |
| TIL | TPP1 | 0.537 | 0.00E+00 |
| TIL | HSPA12B | 0.537 | 1.57E-31 |
| TIL | HSPA7 | 0.537 | 1.58E-31 |
| TIL | AC034105.1 | 0.537 | 1.60E-31 |
| TIL | COTL1 | 0.537 | 0.00E+00 |
| TIL | CASP1 | 0.536 | 1.74E-31 |
| TIL | RGS13 | 0.536 | 1.82E-31 |
| TIL | TGFBI | 0.536 | 0.00E+00 |
| TIL | LRRC15 | 0.536 | 1.95E-31 |
| TIL | XCL2 | 0.536 | 2.06E-31 |
| TIL | PRDM1 | 0.536 | 2.13E-31 |
| TIL | KLHDC7B-DT | 0.536 | 2.19E-31 |
| TIL | JAK2 | 0.536 | 2.25E-31 |
| TIL | GNG8 | 0.535 | 2.62E-31 |
| TIL | POSTN | 0.535 | 0.00E+00 |
| TIL | CDH11 | 0.535 | 0.00E+00 |
| TIL | TRBV11-3 | 0.535 | 2.86E-31 |
| TIL | PLCL1 | 0.535 | 3.07E-31 |
| TIL | DSE | 0.534 | 3.28E-31 |
| TIL | ST6GAL1 | 0.534 | 3.57E-31 |
| TIL | HLA-H | 0.534 | 0.00E+00 |
| TIL | IGHA2 | 0.534 | 0.00E+00 |
| TIL | FNBP1 | 0.534 | 0.00E+00 |
| TIL | ADPGK-AS1 | 0.534 | 3.90E-31 |
| TIL | LRRC2 | 0.534 | 3.90E-31 |
| TIL | GAS6 | 0.534 | 4.00E-31 |
| TIL | AC008964.1 | 0.534 | 4.28E-31 |
| TIL | ROR2 | 0.533 | 4.39E-31 |
| TIL | IGLVI-70 | 0.533 | 4.54E-31 |
| TIL | CETP | 0.533 | 5.32E-31 |
| TIL | DRAM1 | 0.533 | 5.73E-31 |
| TIL | ALDH1L2 | 0.533 | 5.75E-31 |
| TIL | STK32B | 0.533 | 5.75E-31 |
| TIL | AC069209.2 | 0.532 | 5.93E-31 |
| TIL | PRR16 | 0.532 | 6.48E-31 |
| TIL | RSAD2 | 0.532 | 6.73E-31 |
| TIL | LINC01727 | 0.532 | 6.73E-31 |
| TIL | BIRC7 | 0.532 | 7.58E-31 |
| TIL | LUM | 0.531 | 0.00E+00 |
| TIL | ZBTB32 | 0.531 | 8.07E-31 |
| TIL | AOX1 | 0.531 | 8.36E-31 |
| TIL | LINC02773 | 0.531 | 8.67E-31 |
| TIL | COL8A2 | 0.531 | 9.52E-31 |
| TIL | MIR4538 | 0.531 | 1.03E-30 |
| TIL | PTGFR | 0.530 | 1.13E-30 |
| TIL | CFI | 0.530 | 1.14E-30 |
| TIL | AL583785.1 | 0.530 | 1.15E-30 |
| TIL | PDLIM3 | 0.530 | 1.17E-30 |
| TIL | LINC00565 | 0.530 | 1.30E-30 |
| TIL | MEOX2 | 0.530 | 1.40E-30 |
| TIL | CH25H | 0.529 | 1.50E-30 |
| TIL | MRO | 0.529 | 1.65E-30 |
| TIL | PKD2L1 | 0.529 | 1.67E-30 |
| TIL | DPYSL2 | 0.529 | 1.69E-30 |
| TIL | AL592429.1 | 0.529 | 1.78E-30 |
| TIL | CTHRC1 | 0.529 | 0.00E+00 |
| TIL | SIGLEC14 | 0.529 | 1.87E-30 |
| TIL | ADPRH | 0.528 | 1.97E-30 |
| TIL | ARHGAP4 | 0.528 | 2.03E-30 |
| TIL | ECM2 | 0.528 | 2.14E-30 |
| TIL | PLAUR | 0.528 | 0.00E+00 |
| TIL | ST8SIA1 | 0.528 | 2.31E-30 |
| TIL | HDC | 0.528 | 2.33E-30 |
| TIL | CAMK4 | 0.528 | 2.34E-30 |
| TIL | AL592164.1 | 0.528 | 2.50E-30 |
| TIL | MEDAG | 0.527 | 2.72E-30 |
| TIL | ST3GAL6 | 0.527 | 2.74E-30 |
| TIL | TMEM131L | 0.527 | 2.78E-30 |
| TIL | SSTR2 | 0.527 | 2.81E-30 |
| TIL | TMEM119 | 0.527 | 2.83E-30 |
| TIL | HPGDS | 0.527 | 3.06E-30 |
| TIL | RPL4P1 | 0.527 | 3.19E-30 |
| TIL | PRR5L | 0.527 | 3.25E-30 |
| TIL | HGF | 0.527 | 3.29E-30 |
| TIL | CD93 | 0.527 | 3.30E-30 |
| TIL | MRC2 | 0.526 | 0.00E+00 |
| TIL | TMEM255A | 0.526 | 3.82E-30 |
| TIL | C1orf127 | 0.526 | 3.98E-30 |
| TIL | SH2D1B | 0.526 | 4.22E-30 |
| TIL | RECK | 0.525 | 5.03E-30 |
| TIL | WAKMAR2 | 0.525 | 5.17E-30 |
| TIL | BOC | 0.525 | 5.31E-30 |
| TIL | FAM177B | 0.525 | 5.36E-30 |
| TIL | IGKV2D-24 | 0.525 | 5.56E-30 |
| TIL | IGKV6D-21 | 0.525 | 5.62E-30 |
| TIL | AL354743.2 | 0.525 | 5.63E-30 |
| TIL | AC022239.1 | 0.525 | 6.10E-30 |
| TIL | IFI44L | 0.525 | 6.10E-30 |
| TIL | IGFLR1 | 0.525 | 6.13E-30 |
| TIL | C4orf50 | 0.524 | 7.50E-30 |
| TIL | TNFAIP3 | 0.524 | 8.05E-30 |
| TIL | VSTM1 | 0.523 | 8.42E-30 |
| TIL | MOXD1 | 0.523 | 8.87E-30 |
| TIL | HMGB3P32 | 0.523 | 9.00E-30 |
| TIL | DDX58 | 0.523 | 9.49E-30 |
| TIL | SHISAL1 | 0.523 | 1.01E-29 |
| TIL | COL1A2 | 0.523 | 0.00E+00 |
| TIL | LINC02397 | 0.523 | 1.08E-29 |
| TIL | AC244472.1 | 0.523 | 1.11E-29 |
| TIL | IGHV3OR16-15 | 0.522 | 1.35E-29 |
| TIL | CD1C | 0.522 | 1.45E-29 |
| TIL | IL6-AS1 | 0.522 | 1.45E-29 |
| TIL | P4HA3 | 0.522 | 1.46E-29 |
| TIL | SNAI1 | 0.521 | 0.00E+00 |
| TIL | CD300H | 0.521 | 1.73E-29 |
| TIL | FMO2 | 0.521 | 1.75E-29 |
| TIL | IGKV1D-42 | 0.521 | 1.77E-29 |
| TIL | FMO1 | 0.521 | 1.78E-29 |
| TIL | OLFML2B | 0.521 | 0.00E+00 |
| TIL | CXCL16 | 0.521 | 0.00E+00 |
| TIL | ZEB1 | 0.520 | 2.09E-29 |
| TIL | ACSM5 | 0.520 | 2.13E-29 |
| TIL | LINC02829 | 0.520 | 2.26E-29 |
| TIL | TAGLN | 0.520 | 0.00E+00 |
| TIL | COL3A1 | 0.520 | 0.00E+00 |
| TIL | GPX8 | 0.520 | 2.51E-29 |
| TIL | CHRDL2 | 0.520 | 2.59E-29 |
| TIL | AC069368.2 | 0.519 | 2.72E-29 |
| TIL | MAP1LC3C | 0.519 | 2.92E-29 |
| TIL | PSMB8 | 0.519 | 0.00E+00 |
| TIL | FNDC1 | 0.519 | 3.26E-29 |
| TIL | GAS1 | 0.519 | 3.45E-29 |
| TIL | OPTN | 0.518 | 0.00E+00 |
| TIL | RNASE1 | 0.518 | 0.00E+00 |
| TIL | FYN | 0.518 | 4.03E-29 |
| TIL | TNC | 0.518 | 0.00E+00 |
| TIL | ARSI | 0.518 | 4.32E-29 |
| TIL | HAMP | 0.518 | 4.62E-29 |
| TIL | DPP4 | 0.517 | 4.92E-29 |
| TIL | AL031651.2 | 0.517 | 4.94E-29 |
| TIL | SAA1 | 0.517 | 5.24E-29 |
| TIL | ZNF804A | 0.517 | 5.30E-29 |
| TIL | TENT5C | 0.517 | 5.57E-29 |
| TIL | OMD | 0.517 | 5.83E-29 |
| TIL | ADAMTSL1 | 0.517 | 6.04E-29 |
| TIL | AC112721.2 | 0.516 | 6.49E-29 |
| TIL | AL360178.1 | 0.516 | 6.62E-29 |
| TIL | NEFH | 0.516 | 7.01E-29 |
| TIL | IGLV1-41 | 0.516 | 7.63E-29 |
| TIL | GRK5 | 0.516 | 8.09E-29 |
| TIL | MSRB3 | 0.516 | 0.00E+00 |
| TIL | MAMLD1 | 0.515 | 8.35E-29 |
| TIL | KIR3DL1 | 0.515 | 9.10E-29 |
| TIL | TAPBP | 0.515 | 0.00E+00 |
| TIL | SPON2 | 0.515 | 0.00E+00 |
| TIL | CAVIN3 | 0.515 | 9.45E-29 |
| TIL | PDGFRA | 0.515 | 9.64E-29 |
| TIL | ADA2 | 0.515 | 9.98E-29 |
| TIL | TRAV34 | 0.515 | 1.01E-28 |
| TIL | LMCD1 | 0.514 | 1.17E-28 |
| TIL | IGHV1-14 | 0.514 | 1.26E-28 |
| TIL | TMEM26 | 0.514 | 1.27E-28 |
| TIL | RASGRP3 | 0.514 | 1.46E-28 |
| TIL | SP100 | 0.513 | 1.48E-28 |
| TIL | MEOX1 | 0.513 | 1.49E-28 |
| TIL | IGKV1D-17 | 0.513 | 1.73E-28 |
| TIL | RARRES2 | 0.513 | 0.00E+00 |
| TIL | KCNE4 | 0.513 | 1.75E-28 |
| TIL | IGHV7-4-1 | 0.513 | 1.81E-28 |
| TIL | IGHD3-3 | 0.513 | 1.88E-28 |
| TIL | NEK6 | 0.513 | 1.90E-28 |
| TIL | PGBD4P1 | 0.512 | 2.10E-28 |
| TIL | LAMP3 | 0.512 | 2.39E-28 |
| TIL | SFRP4 | 0.511 | 2.60E-28 |
| TIL | AC093627.6 | 0.511 | 2.63E-28 |
| TIL | NKAPL | 0.511 | 2.67E-28 |
| TIL | ITGA5 | 0.511 | 0.00E+00 |
| TIL | CCIN | 0.511 | 2.81E-28 |
| TIL | TRBV13 | 0.511 | 2.83E-28 |
| TIL | AC026369.3 | 0.511 | 2.84E-28 |
| TIL | C3orf80 | 0.511 | 2.86E-28 |
| TIL | SLC43A3 | 0.511 | 2.90E-28 |
| TIL | OLR1 | 0.511 | 3.02E-28 |
| TIL | SLCO5A1 | 0.511 | 3.31E-28 |
| TIL | KLHL5 | 0.511 | 3.33E-28 |
| TIL | AC016074.2 | 0.510 | 3.83E-28 |
| TIL | LINC01825 | 0.510 | 3.88E-28 |
| TIL | LAYN | 0.510 | 4.27E-28 |
| TIL | COL5A2 | 0.509 | 0.00E+00 |
| TIL | LITAF | 0.509 | 0.00E+00 |
| TIL | RASGEF1B | 0.509 | 4.88E-28 |
| TIL | IGLJ3 | 0.509 | 5.35E-28 |
| TIL | EDNRA | 0.509 | 5.80E-28 |
| TIL | CAMK1G | 0.509 | 5.87E-28 |
| TIL | RENBP | 0.509 | 5.90E-28 |
| TIL | SOCS3 | 0.508 | 0.00E+00 |
| TIL | TCTEX1D1 | 0.508 | 6.10E-28 |
| TIL | S1PR1 | 0.508 | 6.12E-28 |
| TIL | ITGB3 | 0.508 | 6.15E-28 |
| TIL | RUNX3-AS1 | 0.508 | 6.22E-28 |
| TIL | TRBV11-1 | 0.508 | 6.38E-28 |
| TIL | DENND3 | 0.508 | 6.44E-28 |
| TIL | OR52K3P | 0.508 | 6.49E-28 |
| TIL | MT1M | 0.508 | 6.90E-28 |
| TIL | ANPEP | 0.508 | 7.20E-28 |
| TIL | LGALS12 | 0.508 | 7.54E-28 |
| TIL | GPR176 | 0.508 | 7.70E-28 |
| TIL | ADAM12 | 0.507 | 0.00E+00 |
| TIL | TLR2 | 0.507 | 0.00E+00 |
| TIL | CHRNA6 | 0.507 | 8.91E-28 |
| TIL | PLN | 0.507 | 9.07E-28 |
| TIL | NIBAN3 | 0.507 | 9.45E-28 |
| TIL | ERMN | 0.507 | 9.50E-28 |
| TIL | TRIM69 | 0.507 | 0.00E+00 |
| TIL | FAM124A | 0.507 | 9.75E-28 |
| TIL | MIR4537 | 0.506 | 1.07E-27 |
| TIL | F2R | 0.506 | 1.08E-27 |
| TIL | LTBP2 | 0.506 | 0.00E+00 |
| TIL | LINC01303 | 0.506 | 1.12E-27 |
| TIL | MLKL | 0.506 | 1.25E-27 |
| TIL | IGLV4-60 | 0.506 | 1.32E-27 |
| TIL | KLF9 | 0.506 | 1.33E-27 |
| TIL | ANGPTL1 | 0.506 | 1.35E-27 |
| TIL | CXCL2 | 0.505 | 1.40E-27 |
| TIL | ADAMTS10 | 0.505 | 1.45E-27 |
| TIL | SLC8A1 | 0.505 | 1.55E-27 |
| TIL | LINC00173 | 0.505 | 1.59E-27 |
| TIL | KCNQ5 | 0.505 | 1.71E-27 |
| TIL | PLA1A | 0.505 | 1.71E-27 |
| TIL | G0S2 | 0.504 | 0.00E+00 |
| TIL | CARD8-AS1 | 0.504 | 2.05E-27 |
| TIL | LINC02555 | 0.504 | 2.10E-27 |
| TIL | IGHV3OR16-6 | 0.504 | 2.19E-27 |
| TIL | AP1S2 | 0.504 | 2.25E-27 |
| TIL | CASP1P2 | 0.504 | 2.33E-27 |
| TIL | FN1 | 0.503 | 0.00E+00 |
| TIL | BTN3A2 | 0.503 | 0.00E+00 |
| TIL | MIR3142HG | 0.502 | 3.13E-27 |
| TIL | APCDD1L | 0.502 | 3.21E-27 |
| TIL | GALNT17 | 0.502 | 3.41E-27 |
| TIL | PARP9 | 0.502 | 3.49E-27 |
| TIL | LINC01050 | 0.502 | 3.60E-27 |
| TIL | COL1A1 | 0.502 | 0.00E+00 |
| TIL | PMP22 | 0.502 | 0.00E+00 |
| TIL | IGHV1-17 | 0.502 | 3.70E-27 |
| TIL | KCNJ8 | 0.502 | 3.90E-27 |
| TIL | GALNT10 | 0.502 | 0.00E+00 |
| TIL | AC007991.2 | 0.501 | 4.46E-27 |
| TIL | KIR2DL1 | 0.501 | 4.47E-27 |
| TIL | ADAMTS12 | 0.501 | 4.51E-27 |
| TIL | GADD45B | 0.501 | 4.57E-27 |
| TIL | LINC02642 | 0.501 | 4.71E-27 |
| TIL | AL356417.3 | 0.501 | 4.83E-27 |
| TIL | ACTN1 | 0.501 | 0.00E+00 |
| TIL | IGKV1OR2-3 | 0.501 | 4.99E-27 |
| TIL | ATP8B2 | 0.501 | 5.07E-27 |
| TIL | SNAI3 | 0.501 | 5.12E-27 |
| TIL | AC021683.5 | 0.500 | 5.73E-27 |
| TIL | PALLD | 0.500 | 0.00E+00 |
| TIL | AADAT | -0.502 | 0.00E+00 |
| TIL | KIAA0895L | -0.507 | 8.63E-28 |
| TIL | SRCIN1 | -0.511 | 3.26E-28 |
| TIL | NELFA | -0.524 | 7.07E-30 |
| TIL | CASKIN1 | -0.538 | 1.19E-31 |

**TableS2.** Differentially expressed genes between the high-risk subgroup and the low-risk subgroup.

| Gene | log2 fold change | False discovery rate |
| --- | --- | --- |
| KRTAP3-1 | 4.488 | 0.001 |
| NOTUM | 3.968 | 0.010 |
| CGA | 3.935 | 0.018 |
| FGF19 | 3.400 | 0.033 |
| CGB5 | 3.216 | 0.050 |
| CGB8 | 2.948 | 0.033 |
| CLDN6 | 2.881 | 0.000 |
| KRT34 | 2.836 | 0.006 |
| C6orf15 | 2.612 | 0.003 |
| IGFL2 | 2.520 | 0.000 |
| APCDD1L | 2.494 | 0.000 |
| BEX1 | 2.369 | 0.000 |
| PCP4 | 2.292 | 0.002 |
| EPYC | 2.193 | 0.000 |
| OXTR | 2.139 | 0.017 |
| EFEMP1 | 2.061 | 0.000 |
| SERPINB7 | 1.955 | 0.000 |
| DES | 1.933 | 0.000 |
| LAMP5 | 1.922 | 0.000 |
| KCNH2 | 1.900 | 0.001 |
| ACTC1 | 1.872 | 0.000 |
| ACTG2 | 1.835 | 0.000 |
| COL10A1 | 1.833 | 0.000 |
| CHGA | 1.826 | 0.000 |
| CALB2 | 1.779 | 0.000 |
| HSPB6 | 1.747 | 0.000 |
| PTGIS | 1.734 | 0.000 |
| CASQ2 | 1.733 | 0.000 |
| HSPB7 | 1.678 | 0.000 |
| CNN1 | 1.675 | 0.000 |
| MYH11 | 1.657 | 0.002 |
| SFRP2 | 1.648 | 0.000 |
| AL161431.1 | 1.648 | 0.000 |
| SFRP1 | 1.638 | 0.000 |
| SFRP4 | 1.624 | 0.000 |
| SUGCT | 1.623 | 0.000 |
| COMP | 1.606 | 0.000 |
| FNDC1 | 1.605 | 0.000 |
| EFHD1 | 1.595 | 0.006 |
| LRRC15 | 1.594 | 0.000 |
| SYNM | 1.593 | 0.001 |
| FIBIN | 1.587 | 0.000 |
| SYNDIG1 | 1.569 | 0.000 |
| IGFBPL1 | 1.558 | 0.000 |
| KLK7 | 1.553 | 0.000 |
| KLK8 | 1.545 | 0.000 |
| CSDC2 | 1.515 | 0.000 |
| AC025580.1 | 1.510 | 0.020 |
| ASPN | 1.505 | 0.000 |
| RBP1 | 1.491 | 0.000 |
| PTX3 | 1.490 | 0.002 |
| CHI3L1 | 1.490 | 0.000 |
| MFAP5 | 1.487 | 0.000 |
| GFRA3 | 1.466 | 0.000 |
| CHRDL2 | 1.461 | 0.000 |
| PNMA8A | 1.457 | 0.000 |
| LMOD1 | 1.445 | 0.000 |
| PLN | 1.433 | 0.000 |
| SOSTDC1 | 1.430 | 0.015 |
| NEURL1 | 1.421 | 0.020 |
| PLPP4 | 1.419 | 0.000 |
| TAGLN | 1.411 | 0.000 |
| AC005180.2 | 1.400 | 0.002 |
| COL11A1 | 1.386 | 0.000 |
| MT1A | 1.364 | 0.007 |
| UCHL1 | 1.360 | 0.000 |
| FGFR1 | 1.357 | 0.000 |
| CCN5 | 1.354 | 0.000 |
| FLNC | 1.345 | 0.000 |
| PSD | 1.335 | 0.000 |
| LYVE1 | 1.332 | 0.005 |
| CNTN1 | 1.327 | 0.000 |
| SYNPO2 | 1.319 | 0.008 |
| MIR145 | 1.319 | 0.021 |
| MMP11 | 1.308 | 0.000 |
| ITGA11 | 1.307 | 0.000 |
| SORBS1 | 1.303 | 0.001 |
| CPXM1 | 1.293 | 0.000 |
| SGCA | 1.289 | 0.002 |
| ACTA2 | 1.274 | 0.000 |
| TCEAL7 | 1.272 | 0.000 |
| SSC5D | 1.265 | 0.000 |
| CGREF1 | 1.259 | 0.000 |
| COL3A1 | 1.258 | 0.000 |
| PTN | 1.255 | 0.000 |
| MYLK | 1.250 | 0.000 |
| GFPT2 | 1.249 | 0.000 |
| FBN2 | 1.247 | 0.000 |
| CPXM2 | 1.247 | 0.000 |
| P2RX1 | 1.246 | 0.008 |
| COL1A1 | 1.245 | 0.000 |
| SCGB1A1 | 1.245 | 0.026 |
| SMOC2 | 1.236 | 0.000 |
| PTHLH | 1.236 | 0.000 |
| P4HA3 | 1.227 | 0.000 |
| OGN | 1.219 | 0.000 |
| COL5A1 | 1.218 | 0.000 |
| COL1A2 | 1.217 | 0.000 |
| DACT3 | 1.212 | 0.000 |
| CCDC80 | 1.210 | 0.000 |
| SERPINB2 | 1.209 | 0.001 |
| MSRB3 | 1.202 | 0.000 |
| PDLIM3 | 1.198 | 0.000 |
| ADAM33 | 1.197 | 0.000 |
| CRMP1 | 1.192 | 0.001 |
| CLIC3 | 1.192 | 0.001 |
| CDH11 | 1.189 | 0.000 |
| ISLR | 1.188 | 0.000 |
| AEBP1 | 1.188 | 0.000 |
| PXDN | 1.188 | 0.000 |
| NTM | 1.184 | 0.000 |
| CRYAB | 1.184 | 0.000 |
| DPYSL3 | 1.183 | 0.000 |
| GXYLT2 | 1.178 | 0.000 |
| SORCS2 | 1.178 | 0.000 |
| GAS1 | 1.177 | 0.000 |
| RBP7 | 1.177 | 0.001 |
| FHL1 | 1.175 | 0.000 |
| CTSV | 1.175 | 0.000 |
| TWIST2 | 1.168 | 0.000 |
| DIO2 | 1.167 | 0.000 |
| ISM1 | 1.165 | 0.000 |
| DPT | 1.160 | 0.000 |
| COL6A3 | 1.159 | 0.000 |
| FGF7 | 1.156 | 0.000 |
| PRELP | 1.152 | 0.000 |
| LY6G6C | 1.148 | 0.022 |
| LUM | 1.142 | 0.000 |
| MYL9 | 1.141 | 0.000 |
| JPH2 | 1.139 | 0.000 |
| REEP2 | 1.136 | 0.000 |
| DCN | 1.135 | 0.000 |
| FAP | 1.134 | 0.000 |
| JAM3 | 1.131 | 0.000 |
| ADAMTS12 | 1.131 | 0.000 |
| CILP | 1.130 | 0.000 |
| RGS4 | 1.123 | 0.000 |
| SCARF2 | 1.119 | 0.000 |
| DPYSL4 | 1.116 | 0.001 |
| SULF2 | 1.115 | 0.000 |
| PGM5 | 1.113 | 0.009 |
| WNT7A | 1.112 | 0.003 |
| COLEC12 | 1.112 | 0.000 |
| KLK13 | 1.111 | 0.030 |
| TPM2 | 1.111 | 0.000 |
| ADAM12 | 1.109 | 0.000 |
| ARSI | 1.105 | 0.000 |
| MRGPRF | 1.103 | 0.000 |
| COL8A2 | 1.101 | 0.000 |
| CACNA2D1 | 1.096 | 0.000 |
| GNG4 | 1.089 | 0.000 |
| VCAN | 1.089 | 0.000 |
| SULF1 | 1.089 | 0.000 |
| PODN | 1.083 | 0.000 |
| SMTNL2 | 1.081 | 0.017 |
| THBS2 | 1.081 | 0.000 |
| RRAD | 1.078 | 0.000 |
| DACT1 | 1.076 | 0.000 |
| COL12A1 | 1.076 | 0.000 |
| EREG | 1.074 | 0.001 |
| CILP2 | 1.071 | 0.000 |
| FBN1 | 1.059 | 0.000 |
| TPM1 | 1.052 | 0.000 |
| SPON1 | 1.047 | 0.000 |
| SCT | 1.046 | 0.022 |
| KCNMB1 | 1.045 | 0.005 |
| RPLP0P2 | 1.042 | 0.000 |
| CTHRC1 | 1.040 | 0.000 |
| RBPMS2 | 1.038 | 0.000 |
| COL5A2 | 1.037 | 0.000 |
| COL8A1 | 1.028 | 0.000 |
| ANPEP | 1.027 | 0.007 |
| POSTN | 1.025 | 0.000 |
| AHNAK2 | 1.022 | 0.000 |
| WNT2 | 1.016 | 0.000 |
| TIMP2 | 1.013 | 0.000 |
| C7 | 1.013 | 0.003 |
| PODNL1 | 1.011 | 0.000 |
| CLDN11 | 1.011 | 0.001 |
| CLIP3 | 1.006 | 0.000 |
| SLC16A2 | 1.004 | 0.000 |
| CTSG | 1.004 | 0.009 |
| ABCA4 | 1.000 | 0.003 |
| RBP5 | -1.001 | 0.000 |
| ZAP70 | -1.003 | 0.000 |
| AC007130.1 | -1.005 | 0.046 |
| FBP1 | -1.005 | 0.000 |
| LGALS4 | -1.006 | 0.000 |
| SLITRK6 | -1.007 | 0.008 |
| TIGIT | -1.011 | 0.018 |
| AQP3 | -1.013 | 0.000 |
| ACSM6 | -1.015 | 0.006 |
| NAPSB | -1.019 | 0.037 |
| CD5 | -1.025 | 0.004 |
| APOL1 | -1.033 | 0.000 |
| MIR200B | -1.041 | 0.012 |
| CXCR6 | -1.041 | 0.001 |
| UGT2B7 | -1.043 | 0.035 |
| AL357033.4 | -1.045 | 0.000 |
| TMPRSS4 | -1.075 | 0.000 |
| MAP4K1 | -1.075 | 0.005 |
| TRAC | -1.075 | 0.010 |
| SMAD6 | -1.077 | 0.000 |
| AC019117.2 | -1.079 | 0.000 |
| HOXB8 | -1.088 | 0.014 |
| NSG1 | -1.090 | 0.003 |
| LCK | -1.091 | 0.000 |
| RNU6ATAC18P | -1.093 | 0.000 |
| SEPTIN9-DT | -1.100 | 0.000 |
| PAX8 | -1.108 | 0.038 |
| CD3D | -1.110 | 0.000 |
| CD7 | -1.110 | 0.011 |
| MIR205 | -1.115 | 0.035 |
| BHMT | -1.117 | 0.002 |
| SLAMF7 | -1.123 | 0.000 |
| GJD3 | -1.128 | 0.000 |
| AC010378.1 | -1.131 | 0.016 |
| SLA2 | -1.134 | 0.006 |
| HPGD | -1.135 | 0.000 |
| OR2I1P | -1.138 | 0.009 |
| AC090954.1 | -1.140 | 0.000 |
| GNLY | -1.142 | 0.048 |
| TRBD1 | -1.143 | 0.001 |
| S1PR4 | -1.144 | 0.000 |
| SHROOM1 | -1.145 | 0.000 |
| SPDEF | -1.151 | 0.000 |
| TRBC1 | -1.157 | 0.001 |
| FCMR | -1.158 | 0.000 |
| TRPA1 | -1.159 | 0.023 |
| AC007991.2 | -1.163 | 0.024 |
| SIRPG | -1.173 | 0.000 |
| CD27 | -1.178 | 0.004 |
| MTRNR2L12 | -1.179 | 0.020 |
| HOXB3 | -1.183 | 0.000 |
| ADAM28 | -1.183 | 0.000 |
| CD2 | -1.188 | 0.001 |
| ITGAL | -1.197 | 0.000 |
| TBC1D10C | -1.207 | 0.001 |
| CD3E | -1.216 | 0.002 |
| DUSP2 | -1.216 | 0.000 |
| AL133370.1 | -1.221 | 0.001 |
| HMGCS2 | -1.239 | 0.001 |
| TSPAN8 | -1.246 | 0.000 |
| AC004687.1 | -1.250 | 0.001 |
| CD52 | -1.260 | 0.017 |
| AC133530.1 | -1.263 | 0.011 |
| SLAMF6 | -1.264 | 0.019 |
| MYLK4 | -1.270 | 0.049 |
| CXCR3 | -1.275 | 0.002 |
| AL121790.2 | -1.297 | 0.000 |
| BX571818.1 | -1.298 | 0.000 |
| HOXB5 | -1.307 | 0.000 |
| MYBPC1 | -1.321 | 0.022 |
| SLC14A1 | -1.335 | 0.000 |
| LINC01871 | -1.340 | 0.000 |
| LINC02195 | -1.343 | 0.037 |
| CYP4F12 | -1.348 | 0.000 |
| NKG7 | -1.363 | 0.014 |
| CD8A | -1.366 | 0.007 |
| SIT1 | -1.373 | 0.023 |
| HSD17B2 | -1.375 | 0.000 |
| TRBV7-2 | -1.391 | 0.016 |
| MIR6784 | -1.397 | 0.000 |
| KRTAP5-9 | -1.400 | 0.000 |
| PDCD1 | -1.407 | 0.004 |
| PPM1N | -1.422 | 0.000 |
| HOXB6 | -1.433 | 0.000 |
| PLA2G10 | -1.434 | 0.000 |
| TNFRSF17 | -1.484 | 0.050 |
| RHEX | -1.503 | 0.001 |
| SHH | -1.535 | 0.000 |
| STK32A-AS1 | -1.551 | 0.014 |
| AC023421.2 | -1.582 | 0.000 |
| GZMH | -1.586 | 0.013 |
| BTBD16 | -1.595 | 0.000 |
| KRTAP5-10 | -1.685 | 0.000 |
| ATOH8 | -1.719 | 0.000 |
| LEAP2 | -1.727 | 0.000 |
| CLCA4 | -1.729 | 0.000 |
| TRIM31 | -1.747 | 0.000 |
| ERN2 | -1.754 | 0.000 |
| DUOX2 | -1.764 | 0.001 |
| GZMA | -1.807 | 0.019 |
| SPOCD1 | -1.816 | 0.000 |
| MUC2 | -1.820 | 0.001 |
| REN | -1.825 | 0.014 |
| MOGAT2 | -1.836 | 0.000 |
| LINC00930 | -1.868 | 0.000 |
| ZNF683 | -1.888 | 0.000 |
| HOXB-AS3 | -1.928 | 0.002 |
| TFF1 | -1.958 | 0.000 |
| CYP4F29P | -2.001 | 0.000 |
| ANXA10 | -2.039 | 0.000 |
| AC012307.1 | -2.043 | 0.000 |
| CYP4F8 | -2.066 | 0.008 |
| UGT2B15 | -2.262 | 0.026 |
| LINC02446 | -2.268 | 0.003 |
| AL162376.1 | -2.338 | 0.001 |
| AC078880.3 | -2.366 | 0.000 |
| DUOXA2 | -2.390 | 0.001 |
| CTSE | -2.570 | 0.000 |
| CRTAC1 | -2.955 | 0.001 |
| GKN1 | -2.974 | 0.000 |
| PRSS1 | -3.675 | 0.031 |
| MS4A8 | -4.287 | 0.001 |
